# Supplementary material for: Association between burnout and empathy in medical residents
Source: PLoS One. 2024 Apr 9;19(4):e0301636. doi: 10.1371/journal.pone.0301636 (PMC11003688; doi:10.1371/journal.pone.0301636)
Supplement: Attachment — Submitted filename: PONE-D-23-22729_R2_reviewer _2).pdf [file pone.0301636.s002.pdf]

PLOS ONE

Association between Burnout and Empathy in medical residents

--Manuscript Draft--

**Manuscript Number:**	PONE-D-23-22729R1
**Article Type:**	Research Article
**Full Title:**	Association between Burnout and Empathy in medical residents
**Short Title:**	Association between Burnout and Empathy
**Corresponding Author:**	Abdolhussein shakurnia, MSc<br>Ahvaz Jundishapur University of Medical Sciences: Ahvaz Jondishapour University of Medical Sciences<br>Ahvaz, IRAN, ISLAMIC REPUBLIC OF
**Keywords:**	empathy, burnout, medical residents
**Abstract:**	Background<br>Burnout is a work-related syndrome that can affect physicians' performance. Empathy is also one of the clinical competencies in whose formation many factors play a role. Empathy and burnout are important topics of research in medical sciences and both are related to the communication between the patient and the physician. This study investigated the relationship between occupational burnout and empathy among medical residents.<br>Method<br>This cross-sectional study was conducted on 297 medical residents in Ahvaz Jundishapur University of Medical Sciences from January to March 2021. The data collection tools were the Jefferson Scale of Empathy (JSE) and The Maslach Burnout Inventory (MBI). The reliability of the instruments was measured by calculating Cronbach's alpha. Data were analyzed by SPSS-18, using the Pearson correlation test and linear regression models.<br>Results<br>The average age of the study population was 33.06 ±4.7 years, with more than half being females (57.6%), and married (51.9%). The residents' mean scores of empathy and overall burnout were 102.87 out of 140 and 55.90 out of 132, respectively. The burnout scores showed an inverse correlation with the overall empathy scores ($r = -0.123$, $P = 0.035$), and the score of standing in the patient's shoes ($r = -0.165$, $P = 0.004$). Linear regression test was used to detect which dimension of empathy was a better predictor for the reduction of burnout scores. Results showed that the best predictor was standing in the patient's shoes ($P = 0.014$).<br>Conclusion<br>The findings showed a negative association between empathy and burnout among medical residents, which suggests the need for adjustment of the existing burnout management at the institutional level. Therefore, residents should be supported by managers to reduce burnout and improve their empathic behavior.
**Order of Authors:**	Mehrnoosh Zakerkish
	Abdolhussein shakurnia, MSc
	Ali Hafezi
	Mahmood Maniati
**Response to Reviewers:**	ACADEMIC EDITOR:<br>- Please provide strong justification of the importance of your research and difference between it and other similar studies specially in your country.<br><br>According to the reviewer's comment, corrections were made in the final paragraph of the introduction section.<br><br>- The study design: it's known that the observational correlational studies include; cross sectional studies, case control studies, cohort studies. As your study is suitable to be crooss sectional study design, so I prefere to be more specific in mentioning the study design as cross section study.

In the manuscript, the study design was to cross-sectional.

- The method part need to be more oraganized as; study design, timing and setting, participant, sampling, tools of data collection, ethical consideration and data mangment and analysis.

The method section was organized according to the reviewer's comment.

- Please mention the response rate of the participants in the method section.

According to the reviewer recommendation, the response rate was transferred to the method section.

- Did you conduct a pilot study?

No

==============================

Journal Requirements:
When submitting your revision, we need you to address these additional requirements.
1. Please ensure that your manuscript meets PLOS ONE's style requirements, including those for file naming. The PLOS ONE style templates can be found at https://journals.plos.org/plosone/s/file?id=wjVg/PLOSOne_formatting_sample_main_body.pdf and https://journals.plos.org/plosone/s/file?id=ba62/PLOSOne_formatting_sample_title_authors_affiliations.pdf.
Our manuscript meets PLOS ONE's style requirements, including those for file naming. The PLOS ONE style templates.
2. Please provide additional details regarding participant consent. In the ethics statement in the Methods and online submission information, please ensure that you have specified what type you obtained (for instance, written or verbal, and if verbal, how it was documented and witnessed). If your study included minors, state whether you obtained consent from parents or guardians. If the need for consent was waived by the ethics committee, please include this information.
The data were collected in a hospital following relevant guidelines and using a paper-based process. As far as the paper-based method was concerned, a research assistant handed the participants an information sheet delineating the study's rationale and the allotted time to complete the survey. They had 10 minutes to decide whether they were willing to collaborate in the study or not. If they were, the research assistant would hand them the questionnaires. Participation in the study was voluntary, and there was no compensation for completing the questionnaire. Adhering to the policy of strict confidentiality, the signatures of the participants were not required, and all the participants retained the right to withdraw from the research at any time without giving any reason.
3. In your Data Availability statement, you have not specified where the minimal data set underlying the results described in your manuscript can be found. PLOS defines a study's minimal data set as the underlying data used to reach the conclusions drawn in the manuscript and any additional data required to replicate the reported study findings in their entirety. All PLOS journals require that the minimal data set be made fully available. For more information about our data policy, please see http://journals.plos.org/plosone/s/data-availability.
All data are available by corresponding author via Email (shakurnia@yahoo.com) upon reasonable request.
"Upon re-submitting your revised manuscript, please upload your study's minimal underlying data set as either Supporting Information files or to a stable, public repository and include the relevant URLs, DOIs, or accession numbers within your revised cover letter. For a list of acceptable repositories, please see http://journals.plos.org/plosone/s/data-availability#loc-recommended-repositories. Any potentially identifying patient information must be fully anonymized.
Important: If there are ethical or legal restrictions to sharing your data publicly, please explain these restrictions in detail. Please see our guidelines for more information on what we consider unacceptable restrictions to publicly sharing data: http://journals.plos.org/plosone/s/data-availability#loc-unacceptable-data-access-restrictions. Note that it is not acceptable for the authors to be the sole named

individuals responsible for ensuring data access.
We will update your Data Availability statement to reflect the information you provide in your cover letter.

Reviewers' comments:

Reviewer's Responses to Questions
Comments to the Author

1. Is the manuscript technically sound, and do the data support the conclusions?

The manuscript must describe a technically sound piece of scientific research with data that supports the conclusions. Experiments must have been conducted rigorously, with appropriate controls, replication, and sample sizes. The conclusions must be drawn appropriately based on the data presented.
Reviewer #1: Partly
Reviewer #2: Partly

____
2. Has the statistical analysis been performed appropriately and rigorously?
Reviewer #1: Yes
Reviewer #2: Yes

____
3. Have the authors made all data underlying the findings in their manuscript fully available?

The PLOS Data policy requires authors to make all data underlying the findings described in their manuscript fully available without restriction, with rare exception (please refer to the Data Availability Statement in the manuscript PDF file). The data should be provided as part of the manuscript or its supporting information, or deposited to a public repository. For example, in addition to summary statistics, the data points behind means, medians and variance measures should be available. If there are restrictions on publicly sharing data—e.g. participant privacy or use of data from a third party—those must be specified.
Reviewer #1: Yes
Reviewer #2: Yes

____
4. Is the manuscript presented in an intelligible fashion and written in standard English?

PLOS ONE does not copyedit accepted manuscripts, so the language in submitted articles must be clear, correct, and unambiguous. Any typographical or grammatical errors should be corrected at revision, so please note any specific errors here.
Reviewer #1: No
Reviewer #2: No

____
5. Review Comments to the Author

Please use the space provided to explain your answers to the questions above. You may also include additional comments for the author, including concerns about dual publication, research ethics, or publication ethics. (Please upload your review as an attachment if it exceeds 20,000 characters)
Reviewer #1: The research question has been widely addressed by recent published literature and fails to add to the existing knowledge base. Examples include:
Pitanupong J, Sathaporn K, Ittasakul P, Karawekpanyawong N. Relationship of mental health and burnout with empathy among medical students in Thailand: A multicenter cross-sectional study. Plos One. 2023 Jan 5;18(1):e0279564.
Picard J, Catu-Pinault A, Boujut E, Botella M, Jaury P, Zenasni F. Burnout, empathy and their relationships: a qualitative study with residents in General Medicine. Psychology, health & medicine. 2016 Apr 2;21(3):354-61.
Thirioux B, Birault F, Jaafari N. Empathy is a protective factor of burnout in physicians: new neuro-phenomenological hypotheses regarding empathy and sympathy in care relationship. Frontiers in psychology. 2016 May 26;7:763.
Amendments were made in the introduction section in order to satisfy the request of

	the reviewer's comment.<br>Reviewer #2: Dear Author, Though it is an interesting research, there is always room for improvement. You may kindly consider the following points:<br>1) Thoroughly revise your manuscript and read your Abstract at two places in your article and make sure that both texts are the same and present in the main article as well. Try to improve your wording.<br>The abstract was reviewed and revised.<br>2)Give further strength to your rationale/purpose of study.<br>The rationale and purpose of the study were reviewed and completed.<br><br>3)In methods, too much detail is given under MEASURES section. Try to add your own performed METHODS rather than giving a literature review. Consider renaming your study design. Mention type of regression used. Exclusion criteria has some not-included variables which may be deleted. If it is a census then there is no need to use the word SAPMLE or sampling technique.<br>In the method section, corrections were made according to the reviewer's comment, and the manuscript was corrected based on the reviewer’s comments.<br><br>4) Reconsider your tables and make it more comprehensible and in standard formats especially table 1 and table 4. Table 3 can be adjusted within table 4. Kindly get guidance from other published researches how to report regression analysis.<br>The tables were revised and Table 3 was merged into Table 4.<br><br>5) Rewrite a short but strong conclusion keeping in view your study objective and results.<br>The conclusion was reviewed and revised in the abstract and in the final paragraph of the manuscript.
**Additional Information:**	
**Question**	**Response**
**Financial Disclosure**<br><br>Enter a financial disclosure statement that describes the sources of funding for the work included in this submission. Review the submission guidelines for detailed requirements. View published research articles from *PLOS ONE* for specific examples.<br><br>This statement is required for submission and **will appear in the published article** if the submission is accepted. Please make sure it is accurate.	The author(s) received no specific funding for this work.

**Unfunded studies**<br>Enter: *The author(s) received no specific funding for this work.*<br><br>**Funded studies**<br>Enter a statement with the following details:<br>• Initials of the authors who received each award<br>• Grant numbers awarded to each author<br>• The full name of each funder<br>• URL of each funder website<br>• Did the sponsors or funders play any role in the study design, data collection and analysis, decision to publish, or preparation of the manuscript?<br>• **NO** - Include this sentence at the end of your statement: *The funders had no role in study design, data collection and analysis, decision to publish, or preparation of the manuscript.*<br>• **YES** - Specify the role(s) played.<br><br>* typeset	
**Competing Interests**<br><br>Use the instructions below to enter a competing interest statement for this submission. On behalf of all authors, disclose any competing interests that could be perceived to bias this work—acknowledging all financial support and any other relevant financial or non-financial competing interests.<br><br>This statement is required for submission and **will appear in the published article** if the submission is accepted. Please make sure it is accurate and that any funding sources listed in your Funding Information later in the submission form are also declared in your Financial Disclosure statement.<br><br>View published research articles from *PLOS ONE* for specific examples.	The authors have declared that no competing interests exist.

**NO authors have competing interests**<br><br>Enter: *The authors have declared that no competing interests exist.*<br><br>**Authors with competing interests**<br><br>Enter competing interest details beginning with this statement:<br><br>*I have read the journal's policy and the authors of this manuscript have the following competing interests: [insert competing interests here]*<br><br>* typeset	
**Ethics Statement**<br><br>Enter an ethics statement for this submission. This statement is required if the study involved:<br><br>• Human participants<br>• Human specimens or tissue<br>• Vertebrate animals or cephalopods<br>• Vertebrate embryos or tissues<br>• Field research<br><br>Write "N/A" if the submission does not require an ethics statement.<br><br>General guidance is provided below. Consult the submission guidelines for detailed instructions. **Make sure that all information entered here is included in the Methods section of the manuscript.**	This study was reviewed and approved by the Ethics Committee of Ahvaz Jundishapur University of Medical Sciences, and its code of ethics was issued (Ethic Code: IR.AJUMS.REC.1399.420).<br>The target group in this study were teaching assistants working in university teaching hospitals who participated in the study voluntarily and completed the questionnaires. By being present at the place and explaining the objectives of the study and obtaining the satisfaction and participation of the participants, the researcher provided the questionnaires to them to complete and deliver.<br>According to the ethical statement, participation in the study was completely voluntary and participants were allowed to withdraw from the study at any time if they did not wish to continue cooperation.

**Format for specific study types**<br><br>**Human Subject Research (involving human participants and/or tissue)**<br>• Give the name of the institutional review board or ethics committee that approved the study<br>• Include the approval number and/or a statement indicating approval of this research<br>• Indicate the form of consent obtained (written/oral) or the reason that consent was not obtained (e.g. the data were analyzed anonymously)<br><br>**Animal Research (involving vertebrate animals, embryos or tissues)**<br>• Provide the name of the Institutional Animal Care and Use Committee (IACUC) or other relevant ethics board that reviewed the study protocol, and indicate whether they approved this research or granted a formal waiver of ethical approval<br>• Include an approval number if one was obtained<br>• If the study involved *non-human primates*, add *additional details* about animal welfare and steps taken to ameliorate suffering<br>• If anesthesia, euthanasia, or any kind of animal sacrifice is part of the study, include briefly which substances and/or methods were applied<br><br>**Field Research**<br><br>Include the following details if this study involves the collection of plant, animal, or other materials from a natural setting:<br>• Field permit number<br>• Name of the institution or relevant body that granted permission	
**Data Availability**<br><br>Authors are required to make all data underlying the findings described fully available, without restriction, and from the time of publication. PLOS allows rare exceptions to address legal and ethical concerns. See the PLOS Data Policy and FAQ for detailed information.	Yes - all data are fully available without restriction

A Data Availability Statement describing where the data can be found is required at submission. Your answers to this question constitute the Data Availability Statement and **will be published in the article**, if accepted.<br><br>**Important:** Stating 'data available on request from the author' is not sufficient. If your data are only available upon request, select 'No' for the first question and explain your exceptional situation in the text box.<br><br>Do the authors confirm that all data underlying the findings described in their manuscript are fully available without restriction?	
**Describe where the data may be found in full sentences. If you are copying our sample text, replace any instances of XXX with the appropriate details.**<br><br>• If the data are **held or will be held in a public repository**, include URLs, accession numbers or DOIs. If this information will only be available after acceptance, indicate this by ticking the box below. For example: *All XXX files are available from the XXX database (accession number(s) XXX, XXX.*).<br>• If the data are all contained **within the manuscript and/or Supporting Information files**, enter the following: *All relevant data are within the manuscript and its Supporting Information files.*<br>• If neither of these applies but you are able to provide **details of access elsewhere**, with or without limitations, please do so. For example:<br><br>*Data cannot be shared publicly because of [XXX]. Data are available from the XXX Institutional Data Access / Ethics Committee (contact via XXX) for researchers who meet the criteria for access to confidential data.*<br><br>*The data underlying the results presented in the study are available from (include the name of the third party*	The dataset/raw data presented in the study is available on request from the Research Development Unit of Ahvaz Jundishapur University of Medical Sciences (Research@ajums.ac.ir) during submission or after publication. The data are not publicly available due to privacy ethics

and contact information or URL).<br>• This text is appropriate if the data are owned by a third party and authors do not have permission to share the data.<br><br>* typeset	
Additional data availability information:	Tick here if your circumstances are not covered by the questions above and you need the journal's help to make your data available.

Powered by Editorial Manager® and ProduXion Manager® from Aries Systems Corporation

Manuscript Click here to access/download;Manuscript;Manuscript.docx

Association between Burnout and Empathy in Medical Residents

Mehrnoosh Zakerkish, Abdolhussein Shakurnia, Ali Hafezi, Mahmood Maniati

Authors' name:

1. Mehrnoosh Zakerkish, (MD), Departments of Internal Medicine, School of medicine, Ahvaz Jundishapur University of Medical Sciences, Ahvaz, Iran. Email: zakerkish-m@ajums.ac.ir
   ORCID: *0000-0003-1680-3329*
2. Abdolhussein Shakurnia, (MSc), Departments of Immunology, School of medicine, Ahvaz Jundishapur University of Medical Sciences, Ahvaz, Iran. Email Address : shakurnia@yahoo.com. ORCID: *0000-0003-3354-2917*
3. Ali Hafezi, (MD), General Physician, School of medicine, Ahvaz Jundishapur University of Medical Sciences, Ahvaz, Iran. Email Address: dr.alihafezi@yahoo.com
   ORCID: *0000-0002-8190-8277*
4. Mahmood Maniati, (PhD), Department of General Courses, School of Medicine, Ahvaz Jundishapur University of Medical Sciences, Ahvaz, Iran. Email Address: maniatim@yahoo.com
   ORCID: 0000-0002-6832-1615

* Correspondence:

Abdolhussein Shakurnia, (MSc), Assistant Professor, Immunology Department. Faculty of medicine, Ahvaz Jundishapur University of Medical Sciences, Ahvaz, Iran. Email: Telephone number: +98 6133330074 . Fax Number: +98 6133332064. Mobile phone number: 09163132602. shakurnia@yahoo.com

****Running Title**: Association between burnout and empathy in medical residents

**** Conflict of interests:** The authors declare no conflict of interests

**** Funding:** No financial support was received by the authors for the present study

**** Acknowledgement:** The authors wish to thank the medical residents who participated in the project and completed the questionnaire.

Author Contributions
Conceptualization: Mehrnoosh Zakerkish, Abdolhussein Shakurnia, Ali Hafezi.
Data curation: Ali Hafezi.
Formal analysis: Abdolhussein Shakurnia, Ali Hafezi.
Investigation: Abdolhussein Shakurnia, Mahmood Maniati.
Project administration: Abdolhussein Shakurnia.
Supervision: Mehrnoosh Zakerkish, Abdolhussein Shakurnia.
Writing – Abdolhussein Shakurnia, Mahmood Maniati.
Writing – review & editing: Mehrnoosh Zakerkish, Abdolhussein Shakurnia, Mahmood Maniati

Abstract

Background

Burnout is a work-related syndrome that can affect physicians' performance. Empathy is also one of the clinical competencies in whose formation many factors play a role. Empathy and burnout are important topics of research in medical sciences and both are related to the communication between the patient and the physician. This study investigated the relationship between occupational burnout and empathy among medical residents.

Method

This cross-sectional study was conducted on 297 medical residents in Ahvaz Jundishapur University of Medical Sciences from January to March 2021. The data collection tools were the Jefferson Scale of Empathy (JSE) and The Maslach Burnout Inventory (MBI). The reliability of the instruments was measured by calculating Cronbach's alpha. Data were analyzed by SPSS-18, using the Pearson correlation test and linear regression models.

Results

The average age of the study population was 33.06 ±4.7 years, with more than half being females (57.6%), and married (51.9%). The residents' mean scores of empathy and overall burnout were 102.87 out of 140 and 55.90 out of 132, respectively. The burnout scores showed an inverse correlation with the overall empathy scores ($r = -0.123$, $P = 0.035$), and the score of standing in the patient's shoes ($r = -0.165$, $P = 0.004$). Linear regression test was used to detect which dimension of empathy was a better predictor for the reduction of burnout scores. Results showed that the best predictor was standing in the patient's shoes ($P = 0.014$).

Conclusion

The findings showed a negative association between empathy and burnout among medical residents, which suggests the need for adjustment of the existing burnout management at the institutional level. Therefore, residents should be supported by managers to reduce burnout and improve their empathic behavior.

Keywords: empathy, burnout, medical residents

Introduction

Medicine is a stressful profession. It is replete with stresses that result from inconsistent and contradictory processes that affect satisfaction and occupational burnout by reducing physical and mental health and occupational involvement. Burnout is a work-related psychological problem that causes emotional problems, reduced personal success, and depersonalization [1]. Occupational burnout is one of the main problems in the quality of life of physicians and health care staff. High levels of burnout in doctors are a threat not only to the doctors themselves but also to their patients and the organizations they work for [2]. Burnout is a multidimensional work-related syndrome that is associated with emotional exhaustion, depersonalization, and diminution of personal accomplishment [3].

Physicians are exposed to heavy workloads, stressful work environments, and high job pressure. These working conditions lead to various types of psychological problems such as anxiety, depression, and workplace burnout in these professionals. These psychological problems have a negative effect on the doctors' quality of life and their satisfaction, which will in turn affect the quality of care they provide to patients [4]. Burnout has become a pervasive problem among medical professionals because can be highly sensitive to levels of empathy [5].

One of the factors related to burnout is empathy. Research has shown that physician empathy is an essential element in health care, which has a great impact on patient satisfaction and adherence to treatment [6]. Empathy is the emotional and cognitive ability to understand the mental states, feelings, thoughts, and motives of others and to respond appropriately to them. Empathy is one of the characteristics of interpersonal behavior that is necessary for effective communication. People with high empathy have more prosocial behaviors while those having low empathy are characterized by more antisocial behaviors. Empathy is an essential element in the physician-patient relationship and affects patient satisfaction and appropriate clinical outcomes [7].

A number of studies have addressed the relationship between empathy and burnout [3, 5, 8]. Ferreira et al., for example, investigated the relationship between empathy and burnout among medical residents and specialists and found that the level of burnout increases with a decrease in empathy, and the aforementioned variable plays a significant role in predicting burnout [6]. Kharidar Atiq also drew the conclusion that there is a negative and significant relationship between empathy and job burnout among nurses [8]. In another research conducted by Yue on medical staff in a hospital in China, a negative relationship was found between empathy and job burnout [7]. According to a systematic review by Wilkinson, the majority of the reviewed studies reported a negative relationship between empathy and job burnout [3]. Of course, the results of some studies have reported a positive relationship between empathy and burnout [2, 9], which needs further investigation.

Medical residents experience a great deal of stress during their training, and this stress eventually decreases their job satisfaction and increases burnout, imposing a large negative impact on the mental health of the residents and the quality of patient care [10, 11]. A number of studies have shown that empathy is one of the factors that protect the mental health of medical staff against burnout [12, 13]. In Iran, the relationship between empathy and burnout has already been

investigated in nurses [8]. However, to the best of our knowledge and review of literature, no previous study has investigated the relationship between empathy and burnout among medical residents in Iran. This study was conducted in order to fulfill this gap in literature. Therefore, given the particular importance of burnout in the mental health and empathic behavior of residents, this study aims to explore the relationship between burnout and empathy among Iranian medical residents. The findings of this study may serve as a foundation for designing mental health interventions, especially those aimed at improving resident empathy.

Method

Study design and participants

This was a cross-sectional investigating the relationship between burnout and empathy in a population of medical residents in Iran. All medical residents studying at Ahvaz Jundishapur University of Medical Sciences (AJUMS) were recruited in this study (n = 472). Of all these residents, 297 completed the questionnaire (the response rate was 63.1 %.). The inclusion criteria were willingness to participate in the study and completing the questionnaire. The exclusion criteria were unwillingness to participate in the study, and inadequate completion of the questionnaires. Data were collected between January 1st and March 30th, 2021. The data were collected in a hospital following relevant guidelines and using a paper-based process. As far as the paper-based method was concerned, a research assistant handed the participants an information sheet delineating the study's rationale and the allotted time to complete the survey. They had 10 minutes to decide whether they were willing to collaborate in the study or not. If they were willing to participate, the research assistant handed them the questionnaires. Participation in the study was voluntary, and there was no compensation for completing the questionnaire. Adhering to the policy of strict confidentiality, the signatures of the participants were not required, and all the participants retained the right to withdraw from the research at any time without giving any reason.

Instruments

All medical residents provided socio-demographic information and completed validated questionnaires on burnout and empathy [9].

Burnout was measured using the Persian version of Maslach Burnout Inventory (MBI). This instrument is a scale composed of 22 items that are scored based on a 7-point Likert scale ranging from 0 (never) to 6 (everyday). The total score of the tool is obtained through the sum of the scores of all items, so the range of the total score is between 0 and 132, with higher scores indicating greater burnout. The scale measures three dimensions: emotional exhaustion, depersonalization, and lack of personal accomplishment [14]. The validity and reliability of this scale have been evaluated in many studies, and it has been shown that this tool and its dimensions have sufficient validity [15–17]. The validity and reliability of this questionnaire have also been evaluated and confirmed in Iran, and its reliability coefficient has been estimated at 0.751 using Cronbach's alpha method [18]. In the present study, the reliability of the questionnaire was calculated by obtaining a Cronbach's alpha of 0.675.

Empathy was measured using the Persian version Jefferson Scale of Physician Empathy (JSPE) The JSPE was developed and standardized by Hojat in 2002 [19]. This instrument is a scale composed of 20 items that are scored based on a 7-point Likert scale ranging from 1 (strongly disagree) to 7 (strongly agree). The total score of the tool is obtained through the sum of the scores of all items, so the range of the total score is between 20 and 140, with higher scores representing greater empathy. This questionnaire has three dimensions: Perspective Taking, Compassionate Care, and Walking in Patient's Shoes. The validity and reliability of this questionnaire have been confirmed in numerous studies [19-21]. In Iran, Shariat et al. reported a Cronbach's alpha coefficient of 0.88 for the Persian version of this questionnaire [21]. In the present study, the reliability of JSPE was calculated obtaining a Cronbach's alpha of 0.766.

Ethical and confidentiality considerations

The study was approved by the Ethics Committee of the Ahvaz Jundishapur University of Medical Sciences (Ref. ID: IR.AJUMS.REC.1399.420). After obtaining the necessary permits for sampling, the researchers went to the teaching hospitals, and after explaining the objectives of the study to the residents and obtaining their consent to participate in the study, the mentioned questionnaires were given to them. Participation in the study was completely optional, the questionnaires were anonymous, and the residents were assured of the confidentiality of their information.

Statistical analysis

The normality of data distribution was checked using the Kolmogorov-Smirnov test which indicated normal distribution of data. The reliability of the MBI and JSPE was measured by calculating Cronbach’s alpha. Data were analyzed using the Statistical Package for the Social Sciences (SPSS) (Version 18.0). We described the numerical data by mean and standard deviation (SD), and nominal data was described as raw counts (n) and percentages (%). The burnout and empathy scores of the residents were calculated. In order to investigate the relationship between burnout and the demographic variables, Pearson's correlation coefficient and linear regression model were used. P values < 0.05 were considered statistically significant.

Results

Demographic characteristics

A total of 297 residents with a mean age of 33.06 ±4.7 years participated in the present study. More than half of the respondents were females (57.6%) and married (51.9%). The mean burnout score of the residents was 55.90±13.25 while their mean empathy score was 102.13±25. The mean scores of burnout and empathy along with their dimensions are displayed in Table 1.

Table 1. Mean scores of burnout and empathy along with their dimensions among medical residents

Variables		Mean	SD	minimum	maximum
**Empathy**		**102.78**	**12.73**	**67**	**134**
dimensions of Empathy	Perspective taking	54.82	7.27	33	70
	Compassionate care	38.32	6.46	18	54
	Walking in the patient's shoes	9.64	2.58	2	14
**Burnout**		**55.90**	**13.25**	**19**	**113**
dimensions of Burnout	Emotional exhaustion (EE)	20.38	11.52	0	51
	Depersonalization (DP)	7.21	5.24	0	24
	Personal accomplishment (PA)	28.36	7.10	4	47

Table 2 shows the correlation coefficients between the residents' mean burnout and empathy scores and between dimensions of burnout and empathy. The results show that there is a significant negative relationship between burnout and empathy (r=-0.123; p=0.035). The correlation coefficients between burnout and empathy dimensions were also negative. However, this relationship was significant only in the sub-scale of "Walking in Patient's Shoes" (r=-0.165; p=0.004). The correlation coefficients of EE and DP dimensions of burnout with empathy and its dimensions were also negative. However, the relationship of PA dimensions of burnout with empathy and its dimensions was positive (Table 2).

Table 2 Correlation matrix between burnout and empathy and between their dimensions

Variable			Empathy	three dimensions of Empathy		
				perspective taking	compassionate care	walking in the patient's shoes
Burnout		r	- 0.123	- 0.110	- 0.052	- 0.165
		p	0.035	00.058	0.37	0.004
three dimensions of Burnout	Emotional Exhaustion	r	-.166**	-.121*	-.139*	-.128*
		p	0.004	00.037	0.016	0.027
	Depersonalization	r	-.378**	-.309**	-.242**	-.385**
		p	0.0001	00.0001	0.0001	0.0001
	Lack of Personal Accomplishment	r	.333**	.230**	.316**	.279**
		p	0.0001	00.0001	0.0001	0.0001

Linear regression was used to investigate the relationship between burnout and empathy and between their dimensions and to see to what extent and in what direction empathy and its dimensions predict burnout. The results of the linear regression equation are given in Table 3. As the findings of this table show, empathy is a negative and significant predictor of burnout.

According to Table 3, the results of linear regression analysis indicate that the explanatory variables (empathy dimensions) can significantly predict and explain changes in the dependent variable (residents' burnout). In other words, the model is meaningful, and among the three dimensions of empathy (i.e., Perspective Taking, Compassionate Care, and Walking in Patient's Shoes) the most determining component is walking in Patient's Shoes. This sub-scale is a negative and significant predictor of job burnout and explains a total of 2.3% of the variance of burnout.

The results also showed that among the three burnout dimensions, emotional exhaustion has the least relationship with empathy dimensions. Also, none of the dimensions of empathy could act as a predictive factor to predict emotional exhaustion in residents. However, the two dimensions of DP and PA could be predicted by the empathy dimensions.

The regression model showed that the empathy dimensions could predict the DP sub-scale of burnout and explain 19.5% of the burnout variance. However, as far as the PA subscale is concerned, empathy dimensions could predict burnout and positively explain 12% of the variance of burnout (Table 3).

Table 3: Predicting burnout through empathy dimensions in residents

Dependent Variable	Predictive Variable	B	Beta	t	P value	R^2	F
Burnout	perspective taking	-0.147	-0.081	-1.252	0.212	0.032	3.264
	compassionate care	0.061	-0.030	0.495	0.647		
	walking in the patient’s shoes	-0.778	-0.152	-2.471	0.014		
Emotional Exhaustion	perspective taking	-0.096	-0.061	-0.942	0.347	0.030	3.048
	compassionate care	-0.156	-0.088	-1.347	0.179		
	standing in the patient’s shoes	-0.373	-0.084	-1.361	0.175		
Depersonalization	perspective taking	-0.141	-0.196	-3.337	0.001	0.195	23.656
	compassionate care	-0.051	-0.063	-1.055	0.292		
	walking in the patient’s shoes	-0.630	-0.310	-5.543	0.0001		

Lack of Personal Accomplishment	perspective taking	0.097	0.100	1.624	0.105	0.120	13.276
	compassionate care	0.268	0.244	3.929	0.0001		
	walking in the patient's shoes	0.268	0.098	1.666	0.097		
Burnout	empathy	-0.128	-0.123	-2.123	0.035	0.015	4.506

Discussion

This study investigated the relationship between empathy and burnout in medical residents. According to our results, the mean empathy score of the residents was 102.78 ± 12.73, with the lowest and highest possible scores of empathy being 20 and 140, respectively. These results indicate that the studied residents' level of empathy is above average, which is in line with the findings of Shariat [21] and Aziz [22], who reported an average level of empathy among the residents in their study. However, in other studies, the level of residents' empathy level has been reported to be lower or higher than average [12, 23, 24]. The inconsistency of these results can be attributed to differences in education and health care systems, and to cultural differences that shape patients' expectations of an "ideal physician."

In this study, the mean burnout score of the studied residents was 55.90±13.25, with the minimum and maximum scores of burnout ranging between 0 and 132. These results indicate that the level of burnout among the residents is lower than average. The literature suggests that many physicians are at risk of burnout or are currently suffering from it. In line with the results of the present study, a systematic review reported the level of burnout in the medical staff to be at an average level [25]. Passos in Brazil reported a high level of burnout among medical residents [26]. In a similar study in Nigeria, a severe degree of burnout in residents was reported [27]. Medical residents face stressful situations during their training, especially when they are in clinical settings. These include acute diseases, accidents, death, and night shifts, which require planning and changes in the curriculum to improve their quality of life and reduce burnout.

In the present study, a negative correlation was found between the empathy and burnout scores. Specifically, an increase in the empathy score led to a decrease in the burnout score. These results are similar to previous reports in other populations [6, 12, 28-30], which means that when physicians are at a high level of burnout, their relationship with their patients seems to worsen. The empathy between the patient and the physicians promotes mutual trust and thus the effectiveness of the treatment. In a study investigating the role of empathy in burnout, Wagman et al. reported that higher levels of empathy are associated with lower levels of burnout [29]. They maintained that given the fact that low empathy can make people more vulnerable to burnout and that strengthening empathy skills can reduce or prevent burnout, it is better to include empathy in medical education programs.

The findings of this study are consistent with those of Yu [7], Khalid [28], and Lee [30] who reported a negative and significant relationship between empathy and burnout. In a systematic

review, Wickenson et al. reported a negative relationship between empathy scores and burnout in most studies conducted on healthcare staff [3]. Wang et al. also reported that empathy has a positive relationship with job satisfaction but a negative association with burnout [4]. To explain this finding, it can be argued that empathy is essential for successful interpersonal functioning, which provides the basis for improving proper relationships with others. If interpersonal relationships and empathy among employees are strengthened, their resilience against difficult situations will automatically increase, and their burnout will experience a downward trend. Examining the relationship between burnout and empathy, Zenasni et al. noted that empathy protects doctors from burnout [31]. According to their study, empathy requires awareness of negative emotions and obliges the doctor to understand patients' mental and psychological conditions through positive interactions. These interactions are resources to deal with stress and burnout at the workplace. Therefore, helping physicians practice more empathy can help protect them from burnout [32].

Despite the compelling evidence indicating a negative relationship between empathy and burnout, there are also studies that have reported a positive correlation between these two variables [3, 9]. This hypothesis is somewhat consistent with Maslach and Jackson's theory according to which empathetic employees are more likely to experience burnout [3]. Considering burnout as a cross-cultural construct, the available evidence emphasizes that the language in which the questionnaire is written may affect the way participants respond to its questions, which will lead to different results. Certainly, further research will shed more light on this topic.

In a systematic review of studies conducted on the relationship between empathy and burnout, Delgado et al. reported that burnout is somewhat associated with depersonalization. In fact, when human aspects of social interactions are reduced, this is likely to lead to a significant reduction in empathy. Hence, burnout is fatal for both the doctor and the patient. Exhausted doctors are less likely to have the ability to stand in the patient's shoes and listen actively. Instead, they prefer to protect themselves by distancing themselves from patients and depersonalizing them. Delgado et al. maintain that the higher the burnout level of physicians, the lower their clinical empathy [13]. Investigating and understanding the complex links between empathy and burnout can help healthcare professionals as well as educational institutions to reduce the risk of burnout.

Our analysis of the relationship between burnout and empathy subscales showed that out of the three empathy subscales, only "Walking in Patient's Shoes" had a significant relationship with burnout, and this subscale can act as a predictive factor and predict burnout in residents. Based on this, residents who had less empathy were associated with higher levels of burnout. In other words, the ability to "walk in the patient's shoes" provides a protective effect for the residents against burnout. Rodríguez-Nogueira et al. suggest that burnout plays a challenging role in clinical empathy and that burnout can affect empathy levels by creating job dissatisfaction [33].

The findings of this study show the discrete importance of different empathy subscales because their impact on burnout subscales is different. Among the three burnout subscales, emotional exhaustion showed the lowest correlation with empathy and its subscales. Examining the relationship between burnout subscales and empathy subscales showed that there is a significant negative relationship between empathy subscales and emotional exhaustion. However, none of the

subscales of empathy has the power to predict emotional exhaustion in the residents. There was a negative and significant relationship between the subscale of depersonalization and empathy and its subscales. Also, the two subscales of "Perspective Taking" and "Walking in Patient's Shoes" could predict the depersonalization subscale in the residents. The results of our regression analysis revealed that these two variables together could predict burnout in a negative direction with a determination coefficient of 19.5% of the variance. Regarding the relationship between the subscale of “lack of personal accomplishment” with empathy and its subscales, it was observed that there is a positive relationship between these variables and that the subscales of "Perspective Taking" and "Compassionate care" could predict the subscale of “lack of personal accomplishment” and explain a total of 12% of the variance. In a systematic review, the relationship of different components of empathy with each component of burnout in doctors was investigated [13]. The results showed that the subscales of emotional exhaustion and depersonalization had a negative relationship with the subscales of perspective-taking, compassionate care, and walking in the patient's shoes. There was also a positive relationship between the subscale of “lack of personal accomplishment” and the subscales of empathy, and high levels of “lack of personal accomplishment” were associated with high levels of “perspective-taking”, “compassionate care”, and “walking in the patient's shoes”, which is consistent with the findings of our study.

Residents should be aware that dealing with patients' pain and suffering on a regular basis puts their emotional health at risk, and that experiencing strong emotions and feelings may affect their personal life. Therefore, having an understanding of burnout and dealing with it as a reality is essential to a resident's career survival. Residents must learn how to protect themselves in this way. A previous study found that residents who had lower empathy scores also had a lower quality of life [34]. Also, in another study, a significant relationship was found between the scores of quality of life and burnout [35]. In a content analysis study, Ahmadian et al. reported that burnout plays a challenging role in clinical empathy [36]. They showed that lack of coping strategies may create a high level of stress and burnout and that extracurricular educational activities, which are considered as coping mechanisms, can have a good effect on mental and physical health and reduce stress and burnout. In order to reduce job burnout and increase the quality of life, planning for extracurricular activities should be done in order to increase coping mechanisms against stress and burnout in students. Managers of residency curricula should be sensitive to the need for the inclusion of training programs for teaching empathic strategies and emotional management for the professional development of medical residents. This will improve the mental health of residents and enhance the quality of medical services by reducing the rate of burnout.

This research had a number of limitations. The main limitation was that this study was conducted only in one university, which limits the generalizability of the results. Another limitation is that data collection was done through convenience sampling, a method that can lead to biases in data collection and sampling. Therefore, caution should be exercised when interpreting the results, paying attention to the fact that the participants in the study were not selected randomly and were not from several universities. Additionally, we did not study other factors that may influence levels of empathy or burnout. These include personality traits, prior psychiatric disorders, or a history of

using medication. Further studies should be conducted to determine other factors influencing empathic capacity during the physician's career.

Conclusion

The findings of this study showed a negative association between empathy and burnout among medical residents, which suggests the need for adjustment of the existing burnout management practices at the institutional level. Of all dimensions of empathy, walking in the patient’s shoes had the most important association with reduced burnout scores and was also a predicting variable for burnout. Medical educators must take these findings into account when planning innovative strategies to promote empathy among medical residents. Therefore, residents should be supported by managers to reduce burnout and improve empathic behavior.

References:

1. Favre S, Bajwa N, Dominicé Dao M, Audétat Voirol M-C, Nendaz M, Junod Perron N, et al. Association between burnout and stigma in physicians. Plos one. 2023; 18(4):e0283556.

2. Hodkinson A, Zhou A, Johnson J, Geraghty K, Riley R, Zhou A, et al. Associations of physician burnout with career engagement and quality of patient care: systematic review and meta-analysis. bmj. 2022; 378.

3. Wilkinson H, Whittington R, Perry L, Eames C. Examining the relationship between burnout and empathy in healthcare professionals: A systematic review. Burnout research. 2017; 6:18-29.

4. Wang Q, Wang L, Shi M, Li X, Liu R, Liu J, et al. Empathy, burnout, life satisfaction, correlations and associated socio-demographic factors among Chinese undergraduate medical students: an exploratory cross-sectional study. BMC medical education. 2019;19:1-10.

5. Pitanupong J, Sathaporn K, Ittasakul P, Karawekpanyawong N. Relationship of mental health and burnout with empathy among medical students in Thailand: A multicenter cross-sectional study. Plos one. 2023; 18(1):e0279564.

6. Ferreira S, Afonso P, Ramos MdR. Empathy and burnout: a multicentre comparative study between residents and specialists. Journal of Evaluation in Clinical Practice. 2020;26(1):216-22.

7. Yue Z, Qin Y, Li Y, Wang J, Nicholas S, Maitland E, et al. Empathy and burnout in medical staff: mediating role of job satisfaction and job commitment. BMC public health. 2022;22(1):1-13.

8. Kharidar S, Zeinali A. The role of emotional and cognitive empathy in predicting job and burnout satisfaction of nurses. Journal of Nursing Education. 2019;7(5):45-51.

9. Maslach C, Jackson SE. The measurement of experienced burnout. Journal of organizational behavior. 1981;2(2):99-113.

10. Maghbouli N, Sohrabpour AA, Fatehi F. The prevalence of burnout in Iranian residents: a cross-sectional study. Future of Medical Education Journal. 2019;9(1):3-8.

11. Zis P, Anagnostopoulos F, Sykioti P. Burnout in medical residents: a study based on the job demands-resources model. The Scientific World Journal. 2014;2014.

12. Bogiatzaki V, Frengidou E, Savakis E, Trigoni M, Galanis P, Anagnostopoulos F. Empathy and burnout of healthcare professionals in public hospitals of Greece. Int J Caring Sci. 2019;12(2):611-26.

13. Delgado N, Delgado J, Betancort M, Bonache H, Harris LT. What is the Link Between Different Components of Empathy and Burnout in Healthcare Professionals? A Systematic Review and Meta-Analysis. Psychology Research and Behavior Management. 2023:447-63.

14. Wickramasinghe ND, Dissanayake DS, Abeywardena GS. Validity and reliability of the Maslach Burnout Inventory-student survey in Sri Lanka. BMC psychology. 2018;6(1):1-10.

15. Al Mutair A, Al Mutairi A, Chagla H, Alawam K, Alsalman K, Ali A. Examining and adapting the psychometric properties of the Maslach burnout inventory-health services survey (MBI-HSS) among healthcare professionals. Applied Sciences. 2020;10(5):1890.

16. Rostami Z, Abedi MR, Schaufeli WB, Ahmadi SA, Sadeghi AH. The psychometric characteristics of Maslach burnout inventory student survey: a study students of Isfahan University. Zahedan Journal of Research in Medical Sciences. 2014;16(9):29-32.

17. Ilic M, Todorovic Z, Jovanovic M, Ilic I. Burnout syndrome among medical students at one University in Serbia: Validity and reliability of the Maslach Burnout Inventory—Student Survey. Behavioral Medicine. 2017;43(4):323-8.

18. Moalemi S, Kavosi Z, Beygi N, Deghan A, Karimi A, Parvizi MM. Evaluation of the Persian version of Maslach burnout inventory-human services survey among Iranian nurses: Validity and reliability. Galen Medical Journal. 2018;7:e995.

19. Hojat M, Gonnella JS, Nasca TJ, Mangione S, Vergare M, Magee M. Physician empathy: definition, components, measurement, and relationship to gender and specialty. American Journal of Psychiatry. 2002;159(9):1563-9.

20. Hojat M, DeSantis J, Shannon SC, Mortensen LH, Speicher MR, Bragan L, et al. The Jefferson Scale of Empathy: a nationwide study of measurement properties, underlying components, latent variable structure, and national norms in medical students. Advances in Health Sciences Education. 2018;23:899-920.

21. Shariat SV, Kaykhavoni A. Empathy in medical residents at Iran University of Medical Sciences. Iranian journal of psychiatry and clinical psychology. 2010;16(3):248-56.

22. Aziz AB, Ali SK. Relationship between level of empathy during residency training and perception of professionalism climate. BMC Medical Education. 2020;20:1-6.

23. Hojat M, Vergare M, Isenberg G, Cohen M, Spandorfer J. Underlying construct of empathy, optimism, and burnout in medical students. International journal of medical education. 2015;6:12.

24. Kusz H, Foreback J, Dohrenwend A. Empathy among internal medicine residents in a community-based training program: a pilot study. MedEdPublish. 2017;6(77):77.

25. Frajerman A, Morvan Y, Krebs M-O, Gorwood P, Chaumette B. Burnout in medical students before residency: a systematic review and meta-analysis. European Psychiatry. 2019;55:36-42.

26. Passos LS, Alves VdS, Rodrigues LARL, Saraiva LV. Burnout syndrome in resident physicians of a Federal University. Revista da Associação Médica Brasileira. 2022;68:24-30.

27. Ogboghodo EO, Edema OM. Assessment of burnout amongst resident doctors in Benin City, Edo State, Nigeria. Nigerian Postgraduate Medical Journal. 2020;27(3):215-23.

28. Khalid MH, Ishtiaq R, Fatima L, Ahmed H, Masood A, Khan TM, et al. Burnout and Empathy: A Cross-Sectional Study among Resident Physicians in Public Sector Hospitals of a Developing Country. European Journal of Medical and Health Sciences. 2021;3(4):121-4.

29. Wagaman MA, Geiger JM, Shockley C, Segal EA. The role of empathy in burnout, compassion satisfaction, and secondary traumatic stress among social workers. Social work. 2015;60(3):201-9.

30. Lee PT, Loh J, Sng G, Tung J, Yeo KK. Empathy and burnout: a study on residents from a Singapore institution. Singapore medical journal. 2018;59(1):50-4.

31. Zenasni F, Boujut E, Woerner A, Sultan S. Burnout and empathy in primary care: three hypotheses. British Journal of General Practice; 2012. p. 346-7.

32. Thirioux B, Birault F, Jaafari N. Empathy is a protective factor of burnout in physicians: new neuro-phenomenological hypotheses regarding empathy and sympathy in care relationship. Frontiers in psychology. 2016;7:763.

33. Rodríguez-Nogueira Ó, Leirós-Rodríguez R, Pinto-Carral A, Álvarez-Álvarez MJ, Fernández-Martínez E, Moreno-Poyato AR. The relationship between burnout and empathy in physiotherapists: a cross-sectional study. Annals of Medicine. 2022;54 (1):933–40.

34. Thomas MR, Dyrbye LN, Huntington JL, Lawson KL, Novotny PJ, Sloan JA, et al. How do distress and well-being relate to medical student empathy? A multicenter study. Journal of general internal medicine. 2007;22:177-83.

35. Paro HB, Silveira PS, Perotta B, Gannam S, Enns SC, Giaxa RR, et al. Empathy among medical students: is there a relation with quality of life and burnout? PloS one. 2014;9(4):e94133.

36. Yazdi NA, Arabshahi KS, Bigdeli S, Ghaffarifar S. Challenges in promoting clinical empathy skills in medical students: A content analysis study. Medical Journal of the Islamic Republic of Iran. 2019;33:104.

Association between Burnout and Empathy in Medical Residents

Mehrnoosh Zakerkish, Abdolhussein Shakurnia, Ali Hafezi, Mahmood Maniati

Authors' name:

1. Mehrnoosh Zakerkish, (MD), Departments of Internal Medicine, School of medicine, Ahvaz Jundishapur University of Medical Sciences, Ahvaz, Iran. Email: zakerkish-m@ajums.ac.ir ORCID: *0000-0003-1680-3329*
2. Abdolhussein Shakurnia, (MSc), Departments of Immunology, School of medicine, Ahvaz Jundishapur University of Medical Sciences, Ahvaz, Iran. Email Address : shakurnia@yahoo.com. ORCID: *0000-0003-3354-2917*
3. Ali Hafezi, (MD), General Physician, School of medicine, Ahvaz Jundishapur University of Medical Sciences, Ahvaz, Iran. Email Address: dr.alihafezi@yahoo.com ORCID: *0000-0002-8190-8277*
4. Mahmood Maniati, (PhD), Department of General Courses, School of Medicine, Ahvaz Jundishapur University of Medical Sciences, Ahvaz, Iran. Email Address: maniatim@yahoo.com ORCID: 0000-0002-6832-1615

* Correspondence:

Abdolhussein Shakurnia, (MSc), Assistant Professor, Immunology Department. Faculty of medicine, Ahvaz Jundishapur University of Medical Sciences, Ahvaz, Iran. Email: Telephone number: +98 6133330074 . Fax Number: +98 6133332064. Mobile phone number: 09163132602. shakurnia@yahoo.com

****Running Title**: Association between burnout and empathy in medical residents

**** Conflict of interests:** The authors declare no conflict of interests

**** Funding:** No financial support was received by the authors for the present study

**** Acknowledgement:** The authors wish to thank the medical residents who participated in the project and completed the questionnaire.

Author Contributions
Conceptualization: Mehrnoosh Zakerkish, Abdolhussein Shakurnia, Ali Hafezi.
Data curation: Ali Hafezi.
Formal analysis: Abdolhussein Shakurnia, Ali Hafezi.
Investigation: Abdolhussein Shakurnia, Mahmood Maniati.
Project administration: Abdolhussein Shakurnia.
Supervision: Mehrnoosh Zakerkish, Abdolhussein Shakurnia.
Writing – Abdolhussein Shakurnia, Mahmood Maniati.
Writing – review & editing: Mehrnoosh Zakerkish, Abdolhussein Shakurnia, Mahmood Maniati

Abstract

Background

Burnout is a work-related syndrome that can affect physicians' performance. Empathy is also one of the clinical competencies~~, and~~ in whose formation many factors play a role~~ in its formation process~~. Empathy and burnout are important topics of research in medical sciences and both are related to the communication between the patient and the physician. ~~In t~~This ~~research,~~study investigated the relationship between occupational burnout and empathy ~~in~~ among medical residents~~ has been investigated~~.

Method

This cross-sectional study was conducted on 297 medical residents in Ahvaz Jundishapur University of Medical Sciences from January to March 2021. The data collection tools were the Jefferson Scale of Empathy (JSE) and The Maslach Burnout Inventory (MBI). The reliability of the instruments was measured by calculating Cronbach’s alpha. Data were analyzed by SPSS-18 ~~software~~, using the Pearson correlation test and linear regression models. ~~In this cross sectional descriptive correlational descriptive study, 473 medical residents in teaching hospitals of Ahvaz Jundishapur University of Medical Sciences in 2021 were recruited, with a convenient sampling method. The Persian version of the Jefferson Scale of Empathy (JSE) and The Maslach Burnout Inventory (MBI) were used to collect data. Data were analyzed by SPSS 18 software, using the Pearson correlation test and linear regression.~~

Results

The average age of the study population was 33.06 ±4.7 years, with more than half ~~were~~being females (57.6%), and married (51.9%).

~~Mean~~The residents’ mean scores of empathy and overall burnout ~~scores~~ were 102.87 out of 140 and 55.90 out of 132, respectively. ~~A score of~~ The burnout scores showed an inverse correlation with the overall empathy scores (r = −0.123, P = 0.035), and the score of standing in the patient’s shoes (r = −0.165, P = 0.004). Linear regression test was used to detect which dimension of

Commented [N1]: According to Reviewers' Comments, this phrase was deleted.

empathy was a better predictor for the reduction of burnout scores. Results showed that the best predictor was standing in the patient’s shoes (P = 0.014). ~~The mean burnout score and the mean empathy score of the medical residents were 55.90±13.25 and 102.7813±12.7325, respectively. Empathy had a negative and significant relationship with burnout (r=-0.123; p=0.035). Also, the empathy and the subscale of "putting oneself in the place of others" could significantly predict residents' burnout.~~

Conclusion

The findings showed a negative association between empathy and burnout among medical residents, which suggests the need for adjustment of the existing burnout management at the institutional level. Therefore, residents ~~may~~should be supported by managers to reduce burnout and improve their empathic behavior. ~~The findings indicated the effective role of empathy in predicting the residents' burnout. Therefore, it is recommended to increase the level of empathy of residents through educational workshops to reduce their burnout.~~

Keywords: empathy, burnout, medical residents

Introduction

Medicine is a stressful profession. It is replete with stresses that result from inconsistent and contradictory processes that affect satisfaction and occupational burnout by reducing physical and mental health and occupational involvement. Burnout is a work-related psychological problem that causes emotional problems, reduced personal success, and depersonalization [1]. Occupational burnout is one of the main problems in the quality of life of physicians ~~doctors~~ and health care staff. High levels of burnout in doctors are a threat not only to the doctors themselves~~, as well as~~ but also to their patients and the organizations they work for [2]. Burnout is a multidimensional work-related syndrome that is associated with emotional exhaustion, depersonalization, and diminution of personal accomplishment [3].

Physicians are exposed to heavy workloads, stressful work environments, and high job pressure. These working conditions lead to various types of psychological problems such as anxiety, depression, and ~~job~~ workplace burnout in these professionals. These psychological problems have a negative effect on the doctors’ quality of life and their satisfaction ~~of doctors' lives~~, which ~~in turn~~

will in turn affect the quality of care they provide~~d~~ to patients [4]. Burnout has become a pervasive problem among medical professionals because ~~burnout it~~ can be highly sensitive to levels of empathy [5].

One of the factors related to burnout is empathy. Research has shown that physician empathy is an essential element in health care, which has a great impact on patient satisfaction and adherence to treatment [6]. Empathy is the emotional and cognitive ability to~~of~~ understand~~ing~~ the mental states, ~~of~~ feelings, thoughts, and motives of others and to respond~~ing~~ appropriately to them. Empathy is one of the characteristics of interpersonal behavior that is necessary for effective communication. People with high empathy have more prosocial behaviors while those having low empathy are characterized by more antisocial behaviors. Empathy is an essential element in the physician-patient relationship and affects patient satisfaction and appropriate clinical outcomes [7].

A number of s~~Several s~~tudies have ~~been done on~~addressed the relationship between empathy and ~~job~~ burnout [3, 5, 8]. ~~For example,~~ Ferreira et al., for example, investigated the relationship between empathy and burnout ~~in~~ among medical residents and specialists and found that the level of burnout increases with a decrease in empathy, and the aforementioned variable plays a significant role in predicting burnout [6]. ~~In a research,~~ Kharidar Atiq also ~~came to~~drew the conclusion that there is a negative and significant relationship between empathy and job burnout ~~of~~ among nurses [8]. In another research conducted by Yue on medical staff in a hospital in China, ~~there was~~ a negative relationship was found between empathy and job burnout [7]. According to a systematic review by Wilkinson, the majority of the reviewed studies reported a negative relationship between empathy and job burnout [3]. Of course, ~~The~~ the results of some studies have ~~shown that there is~~reported a ~~no or~~ positive relationship between empathy and ~~job~~ burnout [2, 9], which needs further investigation.

Medical residents experience a great deal of stress during their training, and this stress eventually ~~causes a decrease in occupation~~decreases their job satisfaction and ~~an~~ increases ~~in~~ burnout, imposing a large negative impact on the mental health of the residents and the quality of patient care [10, 11]. A number of studies have shown that empathy is one of the factors that protect the mental health of medical staff against burnout [12, 13]. In Iran, the relationship between empathy and burnout has already been investigated in nurses [8]. However, to the best of our knowledge ~~and based on our search~~and review of literature, ~~there were~~ no previous stud~~ies~~y ~~concerning~~has investigated the relationship between empathy and burnout among medical residents in Iran. This study was conducted in order to fulfill this ~~research~~ gap in literature. Therefore, given the particular importance of burnout in the mental health and empathic behavior of residents, this study aims to explore the relationship between burnout and empathy among Iranian medical residents. ~~the relationship between these two variables has not been investigated in medical residents. Therefore, given the particular importance of empathy and its role in the mental health of residents, the present study aims to investigate the relationship between empathy and burnout and to determine the role of empathy in predicting the burnout of medical residents.~~The findings of this study may serve as a foundation for designing mental health interventions, especially those aimed at improving resident~~s'~~ empathy.

Method

Study design and Pparticipants

This study was a cross-sectional design, to study the association ofinvestigating the relationship between burnout and empathy in a population of medical residents in Iran. All medical residents studying at Ahvaz Jundishapur University of Medical Sciences (AJUMS) were recruited in theis study (n = 472). Of themall these residents, 297 medical residents completed the questionnaire (the response rate was 63.1%63.1 %.). After approval from the corresponding ethics committees of the Ahvaz Jundishapur University of Medical Sciences=AJUMS (Ethic Code: IR.AJUMS.REC.1399.420), this correlational cross-sectional descriptive study was conducted on all medical residents attending the teaching hospital in AJUMS. All medical residents (n = 472) at AJUMS were recruited in the study. using the convenient sampling method. The inclusion criteria were to study in one of the medical residency courses in AJUMS and a willingness to participate in the study and completing the questionnaireresearch. The exclusion criteria were unwillingness to participate in the study, or failure to complete or and incomplete inadequate completion of the questionnaires.

Data were collected betweenfrom 1st January 1st and2021 to 30th March 30th, 2021. using a convenient sampling method. The data were collected in the givena hospital following relevant guidelines and by using a paper-based process. Concerning As far as the paper-based method was concerned, a research assistant handed them the participants an information sheet delineating the study's rationale and the allotted time to complete the survey. They had 10 minutes to consider decide whether they were willing to collaborate in the study or not. If they wished were willing to participate, the research assistant handed them the questionnaires. Participation in the study was voluntary, and there was no compensation for completing the questionnaire. Adhering to the policy of strict confidentiality, the signatures of the participants were not required, and all the participants retained the right to withdraw from the research at any time without giving any reason.

InstrumentsMeasures

All of the medical residents provided socio-demographic information and completed validated questionnaires on burnout and empathy

The Jefferson Scale of Empathy (JSE) and The Maslach Burnout Inventory (MBI) were used to collect data. MBI was used to measure burnout [9].

Burnout was measured using the Persian version of Maslach Burnout Inventory (MBI). This questionnaire was developed by Christian Maslach and Susan X. Jackson in 1981 with the aim of assessing the individual's experience of burnout. This instrumenttool is a scale composed of has 22 items that are scored based based on a 7seven-point Likert scale ranging from 0 (never) zero to 6 (every day)six. The total score of the tool is obtained through the sum of the scores of all items, so the range of the total scores is between 0 and 132, and awith higher scores means indicating greater burnout. The scale measures three dimensions:: MBI measures emotional exhaustion, depersonalization, and lack of personal accomplishment . The first sub-scale of this questionnaire

Formatted: Superscript

Formatted: Superscript

is emotional exhaustion and it contains 9 items that measure people's feelings, interest, and attitude towards their job. The second sub-scale, depersonalization, includes 5 items that evaluate impersonal and emotionless responses of people towards colleagues and clients. The third sub-scale, lack of personal accomplishment, examines the individual's sense of sufficiency and success in their job in 8 items. By adding up the scores, the scores of each of the three subtests can be calculated. In this questionnaire, a higher score indicates a greater burnout experience [14]. The validity and reliability of this scale have been evaluated in many studies, and it has been shown that this tool and its sub-scalesdimensions have sufficient validity [154–176]. Ilic calculated the reliability coefficient of the burnout scale by obtaining a Cronbach's alpha of 0.869 for emotional exhaustion, 0.856 for depersonalization, 0.852 for lack of personal accomplishment, and 0.757 for the whole questionnaire [17]. The validity and reliability of this questionnaire in Iran have also been evaluated checked and confirmed in Iran, and its reliability coefficient has been estimated at 0.751 using Cronbach's alpha method [18]. In the present study, the reliability of the questionnaire was calculated by obtaining a Cronbach's alpha of 0.675.

Empathy was measured using the Persian version Jefferson Scale of Physician Empathy (JSPE) The JSPE was developed and standardized by Hojat in 2002 [19]. This instrument is a scale composed of 20 items that are scored based on a 7-point Likert scale ranging from 1 (strongly disagree) to 7 (strongly agree). The total score of the tool is obtained through the sum of the scores of all items, so the range of the total scores is between 20 and 140, and awith higher scores meansrepresenting greater empathy.

This questionnaire contains 20 items to which the respondent gives a score from one to seven, based on their level of agreement, with the number one indicating the least agreement and the number seven indicating the highest agreement. The minimum score is 20 and the maximum score is 140. A higher score represents greater empathy with the patient. This questionnaire has three sub-scalesdimensions: Perspective Taking with 10 questions (score range between 10 and 70), Compassionate Care with 8 questions (score range between 8 and 56), and Walking in Patient's Shoes with 2 questions (score range between 2 and 14). The validity and reliability of this questionnaire have been confirmed in numerous studies [19-20, 21]. Hojat et al. investigated and confirmed the validity and reliability of this tool [19]. In Iran, Shariat et al. reported the a Cronbach's alpha coefficient of 0.88 for the Persian version of this questionnaire to be 0.88 and its test-retest reliability coefficient to be 0.94 [21]. In the present study, the reliability of this questionnaireJSPE was calculated obtaining a Cronbach's alpha of 0.766.

Ethical and confidentiality aspectsconsiderations

The study was approved by the eEthics eCommittees of the Ahvaz Jundishapur University of Medical Sciences (Ethic CodeRef. ID: IR.AJUMS.REC.1399.420). After obtaining the necessary permits for sampling, the researchers went to the teaching hospitals; , and after explaining the objectives of the studyresearch to the residents and obtaining their consent to participate in the studyresearch, the mentioned questionnaires were given to them. Participation in the study was completely optional, the questionnaires were anonymous, and the residents were assured of the confidentiality of their information.

Formatted: Font: Bold

Formatted: Font: Bold

Statistical analysis

~~At first, T~~The normality of ~~the~~ data distribution was checked using the Kolmogorov-Smirnov test which indicated ~~the~~ normal distribution of data. The reliability of the MBI and JSPE was measured by calculating Cronbach's alpha. Data were analyzed using the Statistical Package for the Social Sciences (SPSS) (Version 18.0). We described the numerical data ~~utilizing~~ by mean and standard deviation (SD~~);~~), and nominal data was described as raw counts (n) and percentages (%). The burnout and empathy scores of the residents were calculated. In order to investigate the relationship between burnout and the demographic variables, Pearson's correlation coefficient and linear regression model were used. P values < 0.05 were considered statistically significant.

Formatted: Font color: Red

Results

Demographic characteristics

~~The medical residents who completed the questionnaire were 297 of the 472 total residents that were approached; the response rate was 63.1%.~~

A total of 297 residents with a mean age of 33.06 ±4.7 years participated in the present study. More than half of the respondents were females (57.6%)~~,~~ and married (51.9%). ~~Of them, 67 (22.6%) in the first year, 31.3% in the second year, 22.9% in the third year, and 23.2% in the fourth year. Most of them were female (57.6%) and married (51.9%). Overall, their mean age was 33.06 ±4.7 years.~~ The mean burnout score of the residents was 55.90±13.25 while their mean empathy score was 102.13±25. The mean scores of burnout and~~, and~~ empathy ~~and~~ along with their dimensions ~~their sub-scales are~~are displayed~~shown~~ in Table 1.

Table 1. ~~The residents'~~ M~~m~~ean scores of burnout and empathy ~~and~~ along with their ~~sub-scales~~dimensions ~~in~~among medical residents

Variables	Mean	SD	minimum	maximum
**Empathy levels~~(JSPE total score)~~**	**102.78**	**12.73**	**67**	**134**
~~perspective~~ Perspective taking	54.82	7.27	33	70
~~compassionate~~ Compassionate care	38.32	6.46	18	54
~~standing~~ Walking in the patient's shoes	9.64	2.58	2	14

**Burnout levels~~(MBI-HSS)~~**	**55.90**	**13.25**	**19**	**113**
~~emotional~~ Emotional exhaustion (EE)	20.38	11.52	0	51
Depersonalization (DP)	7.21	5.24	0	24
~~personal~~ Personal accomplishment (PA)	28.36	7.10	4	47

Table 2 shows the correlation coefficients between the residents' mean burnout and empathy scores ~~of residents and the mean empathy scores and~~and between ~~their sub-scales~~dimensions of burnout and empathy. The results show that there is a significant negative relationship between burnout and empathy (r=-0.123; p=0.035). The correlation coefficients between burnout and empathy ~~sub-scales~~dimensions were also negative. However, this relationship was significant only in the sub-scale of "Walking in Patient's Shoes" (r=-0.165; p=0.004). The correlation coefficients of EE and DP ~~sub-scales~~dimensions of burnout with empathy and its ~~sub-scales~~dimensions were also negative. However, the relationship of PA dimensions ~~sub-scale~~ of burnout with empathy and its ~~sub-scales~~dimensions was positive (Table 2).

Table 2 Correlation matrix between burnout and empathy and ~~its~~ between their ~~sub-scales~~dimensions

**Variable**			**Empathy**	**three dimensions of Empathy**		
				perspective taking	compassionate care	~~standing~~walking in the patient's shoes
Burnout		r	- 0.123	- 0.110	- 0.052	- 0.165
		p	0.035	00.058	0.37	0.004
		r	-.166**	-.121*	-.139*	-.128*

Formatted: Font: Not Bold

Formatted: Font: Bold

Formatted Table

Formatted: Font: Bold

Formatted: Font: Bold

Formatted: Font: Bold

Formatted Table

three dimensions~~Sub-scales~~ of Burnout	Emotional Exhaustion	p	0.004	00.037	0.016	0.027
	Depersonalization	r	-.378**	-.309**	-.242**	-.385**
		p	0.0001	00.0001	0.0001	0.0001
	Lack of Personal Accomplishment	r	.333**	.230**	.316**	.279**
		p	0.0001	00.0001	0.0001	0.0001

Linear regression was used to investigate the relationship between burnout and empathy and ~~its~~ between their ~~sub-scales~~dimensions and to see to what extent and in what direction empathy and its ~~sub-scales~~dimensions predict burnout. The results of the linear regression equation are given in Table 3. As the findings of ~~Table 3~~this table show, empathy is a negative and significant predictor of burnout.

~~**Table 3: Predicting burnout through the level of empathy in residents**~~

~~Dependent Variable~~	~~Predictors Variable~~	~~B~~	~~Beta~~	~~t~~	~~P-value~~	~~R^2~~	~~F~~
~~Burnout~~	~~empathy~~	~~-0.128~~	~~-0.123~~	~~-2.123~~	~~0.035~~	~~0.015~~	~~4.506~~

According to Table 3~~4~~, the results of linear regression analysis indicate that the explanatory variables (empathy ~~sub-scales~~dimensions) can significantly predict and explain changes in the dependent variable (residents' burnout). In other words, the model is meaningful, and among the three ~~sub-scales~~dimensions of empathy~~,~~ (i.e., Perspective Taking, Compassionate Care, and Walking in Patient's Shoes)~~,~~ the most determining component is ~~walking~~ Walking in Patient's Shoes. This sub-scale is a negative and significant predictor of job burnout and explains a total of 2.3% of the variance of burnout.

The results also showed that among the three burnout ~~sub-scales~~dimensions, emotional exhaustion has the least relationship with empathy ~~sub-scales~~dimensions. Also, none of the ~~sub-scales~~dimensions of empathy could act as a predictive factor to predict emotional exhaustion in residents. However, the two ~~sub-scales~~dimensions of DP and PA could be predicted by the empathy ~~sub-scales~~dimensions.

The regression model showed that the empathy ~~sub-scales~~dimensions could predict the DP sub-scale of burnout and explain 19.5% of the burnout variance. However, as far as the PA subscale is concerned, empathy ~~sub-scales~~dimensions could predict burnout and positively explain 12% of the variance of burnout (Table ~~4~~3).

Table ~~4~~3: Predicting burnout through empathy ~~sub-scales~~dimensions in residents

Formatted: Font: Not Bold

Formatted: Justified

Commented [N2]: According to the Reviewers' comment, Table 3 was deleted and its content was merged into Table 4. Therefore, the title of this consolidated table was considered Table 3.

Dependent Variable	Predictive~~ors~~ Variable	B	Beta	t	P value	R^2	F
Burnout	perspective taking	-0.147	-0.081	-1.252	0.212	0.032	3.264
	compassionate care	0.061	-0.030	0.495	0.647		
	~~standing~~ walking in the patient’s shoes	-0.778	-0.152	-2.471	0.014		
Emotional Exhaustion	perspective taking	-0.096	-0.061	-0.942	0.347	0.030	3.048
	compassionate care	-0.156	-0.088	-1.347	0.179		
	standing in the patient’s shoes	-0.373	-0.084	-1.361	0.175		
Depersonalization	perspective taking	-0.141	-0.196	-3.337	0.001	0.195	23.656
	compassionate care	-0.051	-0.063	-1.055	0.292		
	~~standing~~ walking in the patient’s shoes	-0.630	-0.310	-5.543	0.0001		
Lack of Personal Accomplishment	perspective taking	0.097	0.100	1.624	0.105	0.120	13.276
	compassionate care	0.268	0.244	3.929	0.0001		
	~~standing~~ walking in the patient’s shoes	0.268	0.098	1.666	0.097		
Burnout	empathy	-0.128	-0.123	-2.123	0.035	0.015	4.506

Formatted Table

Discussion

This study investigated the relationship between empathy and burnout in medical residents. According to our results, the mean empathy score of the residents was 102.78 ± 12.73, ~~which varies between~~with the lowest and highest possible scores of empathy being 20 and 140, ~~according to the empathy score range~~respectively. These results indicate that the studied residents' level of empathy is above average, which is in line with the findings of Shariat [21] and Aziz [22], who reported an average level of empathy ~~of~~ among the residents in their study. However, in other studies, the level of residents' empathy level has been reported to be lower or higher than average [12, 23, 24]. The inconsistency of these results can be attributed to differences in education and health care systems, and to cultural differences that ~~determine~~ shape patients' expectations of an "ideal physician."

In this study, the mean burnout score of the studied residents was 55.90±13.25, with the minimum and maximum scores of burnout ranging between 0 and 132. These results indicate that the level of burnout among the residents is lower than average. The literature suggests that many physicians are at risk of burnout or are currently suffering from it. In line with the results of the present study, a systematic review ~~has~~ reported the level of burnout in the medical staff to be at an average level [25]. Passos in Brazil ~~has~~ reported a high level of burnout among medical residents [26]. In a similar study in Nigeria, a severe ~~level~~ degree of burnout in residents ~~has been~~was reported [27]. Medical residents face stressful situations during their training, especially when they are in clinical settings. These include acute diseases, accidents, death, and night shifts, which require planning and changes in the curriculum to improve their quality of life and reduce burnout.

In the present study, a negative correlation was found between the empathy ~~score~~ and ~~the~~ burnout scores. Specifically, ~~with~~ an increase in the empathy score~~,~~ led to a decrease in the burnout score ~~decreased~~. These results are similar to previous reports in other populations [6, 12, 28-30~~].~~], ~~This~~ which means that when physicians are at a high level of burnout, their relationship with their patients seems to ~~become poor~~worsen. The empathy between the patient and the physicians ~~increases~~ promotes mutual trust and thus the effectiveness of the treatment. In a study investigating the role of empathy in burnout, Wagman et al. reported that higher levels of empathy are associated with lower levels of burnout [29]. ~~He~~ They maintained ~~stated~~ that given the fact that low empathy can make people more vulnerable to burnout and that strengthening empathy skills can reduce or prevent burnout, it is better to include empathy in ~~the program of~~ medical education programs.

The findings of this study are consistent with those of Yu [7], Khalid [28], and Lee [30] who reported a negative and significant relationship between empathy and burnout. In a systematic review, Wickenson et al. reported a negative relationship between empathy scores and burnout in most ~~of the~~ studies conducted on healthcare staff [3]. Wang et al. also reported that empathy has a positive relationship with job satisfaction ~~and~~ but a negative association with burnout [4]. ~~In~~ To explain~~ing~~ this finding, it can be argued that empathy is essential for successful interpersonal functioning, which provides the basis for improving proper relationships with others. If interpersonal relationships and empathy among employees are strengthened, their resilience against difficult situations will automatically increase, and their burnout will experience a downward trend. Examining the relationship between burnout and empathy, Zenasni et al. noted that empathy protects doctors from burnout [31]. According to ~~that~~ their study, empathy requires awareness of negative emotions and obliges the doctor to understand patients' mental and psychological conditions through positive interactions. These interactions are resources to deal with stress and burnout at the workplace. Therefore, helping physicians practice more empathy can help~~s~~ protect them from burnout [32].

Despite the compelling evidence indicating a negative relationship between empathy and burnout, there are also studies that have reported a positive correlation between these two variables [3, 9]. This hypothesis is somewhat consistent with Maslach and Jackson's theory ~~that~~ according to which empathetic employees are more likely to experience burnout [3]. Considering burnout as a cross-cultural construct, the available evidence emphasizes that the language ~~of~~ in which the questionnaire is written may affect the way ~~the~~ participants respond~~s~~ to ~~the~~ its questions, which

will lead~~ing~~ to different results. Certainly, ~~more future studies~~further research will shed more light on this topic.

In a systematic review of studies conducted on the relationship between empathy and burnout, Delgado et al. reported that burnout is somewhat associated with depersonalization. In fact, when human aspects of social interactions are reduced, this is likely to lead to a significant reduction in empathy. Hence, burnout is fatal for both the doctor and the patient. Exhausted doctors are less likely to have the ability to stand in the patient's shoes and listen actively. Instead, they prefer to protect themselves by distancing themselves from patients and depersonalizing them. Delgado et al. maintain~~s~~ that the higher the burnout level of physicians, the lower their clinical empathy [13]. Investigating and understanding the complex links between empathy and burnout can help healthcare professionals as well as educational institutions to reduce the risk of burnout.

Our analysis of the relationship between burnout and empathy subscales showed that out of the three empathy subscales, only "Walking in Patient's Shoes" had a significant relationship with burnout, and this subscale can act as a predictive factor ~~could~~ and predict burnout in residents. Based on this, residents who had less empathy ~~had~~ were associated with higher levels of burnout. In other words, the ability to "walk in the patient's shoes" provides a protective effect for the residents against burnout. Rodríguez-Nogueira et al. suggest that burnout plays a challenging role in clinical empathy and that burnout ~~by creating job dissatisfaction~~ can affect empathy levels by creating job dissatisfaction [33].

~~–~~The findings of this study show the discrete importance of different empathy subscales ~~separately~~ because their impact on burnout subscales is different. Among the three burnout subscales, emotional exhaustion showed the lowest correlation with empathy and its subscales. Examining the relationship between burnout subscales and empathy subscales showed that there is a significant negative relationship between empathy subscales and emotional exhaustion. However, none of the subscales of empathy ~~as a predictive factor~~ has the power to predict emotional exhaustion in the residents. There was a negative and significant relationship between the subscale of depersonalization and empathy and its subscales. Also, the two subscales of "Perspective Taking" and "Walking in Patient's Shoes" ~~as predictive factors~~ could predict the depersonalization subscale in the residents. The results of our regression analysis revealed that these two variables ~~combined~~ together could predict burnout in a negative direction with a determination coefficient of 19.5% of the variance. Regarding the relationship between the subscale of ~~reduced personal success~~"lack of personal accomplishment" with empathy and its subscales, it was observed that there is a positive relationship between these variables and that the subscales of "Perspective Taking" and "Compassionate care" ~~are able to~~could predict the subscale of "lack of personal accomplishment"~~reduced personal success,~~ and ~~they~~ explain~~ed~~ a total of 12% of the variance. In a systematic review, the relationship ~~between~~ of different components of empathy ~~and~~ with each component of burnout in doctors was investigated [13]. The results showed that ~~there is a negative relationship between~~ the subscales of emotional exhaustion and depersonalization had a negative relationship with the subscales of perspective-taking, compassionate care, and walking in the patient's shoes. There was also a positive relationship between the subscale of "lack of personal accomplishment"~~personal failure~~ and the subscales of empathy, and high levels of ~~reduced~~

~~personal success~~“lack of personal accomplishment” were associated with high levels of “perspective-taking”, “compassionate care”, and “walking in the patient's shoes”, which is consistent with the findings of our study.

Residents should be aware that dealing with patients' pain and suffering on a regular basis puts their emotional health at risk, and that experiencing strong emotions and feelings may affect their personal life. Therefore, having an understanding of burnout and dealing with it as a reality is essential to a resident's career survival. Residents must learn how to protect themselves in this way. A previous study found that residents who had lower empathy scores also had a lower quality of life [34]. Also, in another study, a significant relationship was found between the scores of quality of life and burnout [35]. ~~Ahmadian et al. reported~~ ~~i~~In a content analysis study, Ahmadian et al. reported that burnout plays a challenging role in clinical empathy [36]. They showed that ~~a~~ lack of coping strategies may create a high level of stress and burnout and that extracurricular educational activities, which are considered as ~~one of the~~ coping mechanisms, can have a good effect on mental and physical health and reduce stress and burnout. In order to reduce job burnout and increase the quality of life, planning for extracurricular activities should be done in order to increase coping mechanisms ~~for~~ against stress and burnout in students. Managers of residency curricula should be sensitive to the need for the inclusion of training programs for teaching empathic strategies and emotional management for the professional development of medical residents. This will improve the mental health of residents and enhance the quality of medical services by reducing the rate of burnout.

This research had a number of limitations. The main limitation was that ~~it~~ this study was conducted only in one university, which limits the generalizability of the results. Another limitation is that data collection was done through convenience sampling, a method that can lead to biases in data collection and sampling. Therefore, caution should be exercised when interpreting the results, paying attention to the fact that the participants in the study were not selected randomly and were not from several universities. Additionally, we did not study other factors that may influence levels of empathy or burnout. These include personality traits, prior psychiatric disorders, or a history of using medication. Further studies should be conducted to determine other factors influencing empathic capacity during the physician's career.

Conclusion

~~To conclude, t~~The findings of this study showed a negative association between empathy and burnout among medical residents, which suggests the need for adjustment of the existing burnout management practices at the institutional level. ~~Among~~Of all ~~of the~~ dimension~~subscale~~s of empathy, ~~standing~~walking in the patient’s shoes h~~eld~~ad the most important association with ~~decreasing~~reduced burnout scores and was also a predicting variable for burnout. Medical educators must take these findings into account when planning innovative strategies to ~~develop~~promote empathy among medical residents. Therefore, residents should be supported by managers to reduce burnout and improve empathic behavior.

Formatted: Font: Not Bold
Formatted: Font: Not Bold
Formatted: Font: Not Bold
Formatted: Font: Not Bold
Formatted: Font: Not Bold
Formatted: Font: Not Bold
Formatted: Font: Not Bold
Formatted: Font: Not Bold
Formatted: Font: Not Bold
Formatted: Font: Not Bold
Formatted: Font: Not Bold
Formatted: Font: Not Bold
Formatted: Font: Not Bold

The level of clinical empathy in the studied medical residents was relatively favorable, and their rate of burnout was below average. We found that there is a negative relationship between empathy and burnout and that empathy can predict the occurrence of burnout in medical residents. Therefore, it can be deduced that high levels of empathy can protect against burnout. When burnout is present, it may not allow for a fully empathic therapeutic relationship with patients. Hence, enhancing empathy skills through training programs can help residents protect themselves from burnout. Therefore, officials and planners of residency education should include the promotion of empathy in the residency curriculum in order to reduce burnout. Examining and understanding the complex links between empathy and burnout can help healthcare professionals reduce the risk of burnout. Future research to identify factors affecting empathy and burnout in residents can be used to design and implement appropriate interventions to reduce burnout in these individuals.

References:

1. Favre S, Bajwa N, Dominicé Dao M, Audétat Voirol M-C, Nendaz M, Junod Perron N, et al. Association between burnout and stigma in physicians. Plos one. 2023; 18(4):e0283556.

2. Hodkinson A, Zhou A, Johnson J, Geraghty K, Riley R, Zhou A, et al. Associations of physician burnout with career engagement and quality of patient care: systematic review and meta-analysis. bmj. 2022; 378.

3. Wilkinson H, Whittington R, Perry L, Eames C. Examining the relationship between burnout and empathy in healthcare professionals: A systematic review. Burnout research. 2017; 6:18-29.

4. Wang Q, Wang L, Shi M, Li X, Liu R, Liu J, et al. Empathy, burnout, life satisfaction, correlations and associated socio-demographic factors among Chinese undergraduate medical students: an exploratory cross-sectional study. BMC medical education. 2019;19:1-10.

5. Pitanupong J, Sathaporn K, Ittasakul P, Karawekpanyawong N. Relationship of mental health and burnout with empathy among medical students in Thailand: A multicenter cross-sectional study. Plos one. 2023; 18(1):e0279564.

6. Ferreira S, Afonso P, Ramos MdR. Empathy and burnout: a multicentre comparative study between residents and specialists. Journal of Evaluation in Clinical Practice. 2020;26(1):216-22.

7. Yue Z, Qin Y, Li Y, Wang J, Nicholas S, Maitland E, et al. Empathy and burnout in medical staff: mediating role of job satisfaction and job commitment. BMC public health. 2022;22(1):1-13.

8. Kharidar S, Zeinali A. The role of emotional and cognitive empathy in predicting job and burnout satisfaction of nurses. Journal of Nursing Education. 2019;7(5):45-51.

9. Maslach C, Jackson SE. The measurement of experienced burnout. Journal of organizational behavior. 1981;2(2):99-113.

10. Maghbouli N, Sohrabpour AA, Fatehi F. The prevalence of burnout in Iranian residents: a cross-sectional study. Future of Medical Education Journal. 2019;9(1):3-8.

11. Zis P, Anagnostopoulos F, Sykioti P. Burnout in medical residents: a study based on the job demands-resources model. The Scientific World Journal. 2014;2014.

12. Bogiatzaki V, Frengidou E, Savakis E, Trigoni M, Galanis P, Anagnostopoulos F. Empathy and burnout of healthcare professionals in public hospitals of Greece. Int J Caring Sci. 2019;12(2):611-26.

13. Delgado N, Delgado J, Betancort M, Bonache H, Harris LT. What is the Link Between Different Components of Empathy and Burnout in Healthcare Professionals? A Systematic Review and Meta-Analysis. Psychology Research and Behavior Management. 2023:447-63.

14. Wickramasinghe ND, Dissanayake DS, Abeywardena GS. Validity and reliability of the Maslach Burnout Inventory-student survey in Sri Lanka. BMC psychology. 2018;6(1):1-10.

15. Al Mutair A, Al Mutairi A, Chagla H, Alawam K, Alsalman K, Ali A. Examining and adapting the psychometric properties of the Maslach burnout inventory-health services survey (MBI-HSS) among healthcare professionals. Applied Sciences. 2020;10(5):1890.

16. Rostami Z, Abedi MR, Schaufeli WB, Ahmadi SA, Sadeghi AH. The psychometric characteristics of Maslach burnout inventory student survey: a study students of Isfahan University. Zahedan Journal of Research in Medical Sciences. 2014;16(9):29-32.

17. Ilic M, Todorovic Z, Jovanovic M, Ilic I. Burnout syndrome among medical students at one University in Serbia: Validity and reliability of the Maslach Burnout Inventory—Student Survey. Behavioral Medicine. 2017;43(4):323-8.

18. Moalemi S, Kavosi Z, Beygi N, Deghan A, Karimi A, Parvizi MM. Evaluation of the Persian version of Maslach burnout inventory-human services survey among Iranian nurses: Validity and reliability. Galen Medical Journal. 2018;7:e995.

19. Hojat M, Gonnella JS, Nasca TJ, Mangione S, Vergare M, Magee M. Physician empathy: definition, components, measurement, and relationship to gender and specialty. American Journal of Psychiatry. 2002;159(9):1563-9.

20. Hojat M, DeSantis J, Shannon SC, Mortensen LH, Speicher MR, Bragan L, et al. The Jefferson Scale of Empathy: a nationwide study of measurement properties, underlying components, latent variable structure, and national norms in medical students. Advances in Health Sciences Education. 2018;23:899-920.

21. Shariat SV, Kaykhavoni A. Empathy in medical residents at Iran University of Medical Sciences. Iranian journal of psychiatry and clinical psychology. 2010;16(3):248-56.

22. Aziz AB, Ali SK. Relationship between level of empathy during residency training and perception of professionalism climate. BMC Medical Education. 2020;20:1-6.

23. Hojat M, Vergare M, Isenberg G, Cohen M, Spandorfer J. Underlying construct of empathy, optimism, and burnout in medical students. International journal of medical education. 2015;6:12.

24. Kusz H, Foreback J, Dohrenwend A. Empathy among internal medicine residents in a community-based training program: a pilot study. MedEdPublish. 2017;6(77):77.

25. Frajerman A, Morvan Y, Krebs M-O, Gorwood P, Chaumette B. Burnout in medical students before residency: a systematic review and meta-analysis. European Psychiatry. 2019;55:36-42.

26. Passos LS, Alves VdS, Rodrigues LARL, Saraiva LV. Burnout syndrome in resident physicians of a Federal University. Revista da Associação Médica Brasileira. 2022;68:24-30.

27. Ogboghodo EO, Edema OM. Assessment of burnout amongst resident doctors in Benin City, Edo State, Nigeria. Nigerian Postgraduate Medical Journal. 2020;27(3):215-23.

28. Khalid MH, Ishtiaq R, Fatima L, Ahmed H, Masood A, Khan TM, et al. Burnout and Empathy: A Cross-Sectional Study among Resident Physicians in Public Sector Hospitals of a Developing Country. European Journal of Medical and Health Sciences. 2021;3(4):121-4.

29. Wagaman MA, Geiger JM, Shockley C, Segal EA. The role of empathy in burnout, compassion satisfaction, and secondary traumatic stress among social workers. Social work. 2015;60(3):201-9.

30. Lee PT, Loh J, Sng G, Tung J, Yeo KK. Empathy and burnout: a study on residents from a Singapore institution. Singapore medical journal. 2018;59(1):50-4.

31. Zenasni F, Boujut E, Woerner A, Sultan S. Burnout and empathy in primary care: three hypotheses. British Journal of General Practice; 2012. p. 346-7.

32. Thirioux B, Birault F, Jaafari N. Empathy is a protective factor of burnout in physicians: new neuro-phenomenological hypotheses regarding empathy and sympathy in care relationship. Frontiers in psychology. 2016;7:763.

33. Rodríguez-Nogueira Ó, Leirós-Rodríguez R, Pinto-Carral A, Álvarez-Álvarez MJ, Fernández-Martínez E, Moreno-Poyato AR. The relationship between burnout and empathy in physiotherapists: a cross-sectional study. Annals of Medicine. 2022;54 (1):933–40.

34. Thomas MR, Dyrbye LN, Huntington JL, Lawson KL, Novotny PJ, Sloan JA, et al. How do distress and well-being relate to medical student empathy? A multicenter study. Journal of general internal medicine. 2007;22:177-83.

35. Paro HB, Silveira PS, Perotta B, Gannam S, Enns SC, Giaxa RR, et al. Empathy among medical students: is there a relation with quality of life and burnout? PloS one. 2014;9(4):e94133.

36. Yazdi NA, Arabshahi KS, Bigdeli S, Ghaffarifar S. Challenges in promoting clinical empathy skills in medical students: A content analysis study. Medical Journal of the Islamic Republic of Iran. 2019;33:104.

Dear Dr. El Seifi,
Academic Editor PLOS ONE

PONE-D-23-22729. Association between Burnout and Empathy in Medical Residents

It is with great pleasure that we resubmit to you a revised version of manuscript entitled "*Association between Burnout and Empathy in Medical Residents*". We would like to thank you for giving us the opportunity to revise and resubmit this manuscript. We appreciate the time and care you and the reviewers took in providing us with the valuable comments, and we have incorporated the suggested changes into the manuscript to the best of our ability. No doubt that these insightful comments have added to the theoretical and practical strength of this manuscript. We are ready to take more suggestions from you should you think the revised manuscript is still in need of further refinement.

Kind regards,
Corresponding author
Abdolhussein Shakurnia

==============================
ACADEMIC EDITOR:
- Please provide strong justification of the importance of your research and difference between it and other similar studies specially in your country.

According to the reviewer's comment, corrections were made in the final paragraph of the introduction section.

- The study design: it's known that the observational correlational studies include; cross sectional studies, case control studies, cohort studies. As your study is suitable to be crooss sectional study design, so I prefere to be more specific in mentioning the study design as cross section study.
In the manuscript, the study design was to cross-sectional.

- The method part need to be more oraganized as; study design, timing and setting, participant, sampling, tools of data collection, ethical consideration and data mangment and analysis.
The method section was organized according to the reviewer's comment.

- Please mention the response rate of the participants in the method section.

According to the reviewer recommendation, the response rate was transferred to the method section.

- Did you conduct a pilot study?
No

================================

Journal Requirements:

When submitting your revision, we need you to address these additional requirements.

1. Please ensure that your manuscript meets PLOS ONE's style requirements, including those for file naming. The PLOS ONE style templates can be found at

https://journals.plos.org/plosone/s/file?id=wjVg/PLOSOne_formatting_sample_main_body.pdf and

https://journals.plos.org/plosone/s/file?id=ba62/PLOSOne_formatting_sample_title_authors_affiliations.pdf.

Our manuscript meets PLOS ONE's style requirements, including those for file naming. The PLOS ONE style templates.

2. Please provide additional details regarding participant consent. In the ethics statement in the Methods and online submission information, please ensure that you have specified what type you obtained (for instance, written or verbal, and if verbal, how it was documented and witnessed). If your study included minors, state whether you obtained consent from parents or guardians. If the need for consent was waived by the ethics committee, please include this information.

The data were collected in a hospital following relevant guidelines and using a paper-based process. As far as the paper-based method was concerned, a research assistant handed the participants an information sheet delineating the study's rationale and the allotted time to complete the survey. They had 10 minutes to decide whether they were willing to collaborate in the study or not. If they were, the research assistant would hand them the questionnaires. Participation in the study was voluntary, and there was no compensation for completing the questionnaire. Adhering to the policy of strict confidentiality, the signatures of the participants were not required, and all the participants retained the right to withdraw from the research at any time without giving any reason.

3. In your Data Availability statement, you have not specified where the minimal data set underlying the results described in your manuscript can be found. PLOS defines a study's minimal data set as the underlying data used to reach the conclusions drawn in the manuscript and any additional data required to replicate the reported study findings in their entirety. All PLOS journals require that the minimal data set be made fully available. For more information about our data policy, please see http://journals.plos.org/plosone/s/data-availability.

All data are available by corresponding author via Email (shakurnia@yahoo.com) upon reasonable request.

"Upon re-submitting your revised manuscript, please upload your study’s minimal underlying data set as either Supporting Information files or to a stable, public repository and include the relevant URLs, DOIs, or accession numbers within your revised cover letter. For a list of acceptable repositories, please see http://journals.plos.org/plosone/s/data-availability#loc-

recommended-repositories. Any potentially identifying patient information must be fully anonymized.

Important: If there are ethical or legal restrictions to sharing your data publicly, please explain these restrictions in detail. Please see our guidelines for more information on what we consider unacceptable restrictions to publicly sharing data: http://journals.plos.org/plosone/s/data-availability#loc-unacceptable-data-access-restrictions. Note that it is not acceptable for the authors to be the sole named individuals responsible for ensuring data access.

We will update your Data Availability statement to reflect the information you provide in your cover letter.

Reviewers' comments:

Reviewer's Responses to Questions

Comments to the Author

1. Is the manuscript technically sound, and do the data support the conclusions?

The manuscript must describe a technically sound piece of scientific research with data that supports the conclusions. Experiments must have been conducted rigorously, with appropriate controls, replication, and sample sizes. The conclusions must be drawn appropriately based on the data presented.

Reviewer #1: Partly

Reviewer #2: Partly

---

2. Has the statistical analysis been performed appropriately and rigorously?

Reviewer #1: Yes

Reviewer #2: Yes

---

3. Have the authors made all data underlying the findings in their manuscript fully available?

The PLOS Data policy requires authors to make all data underlying the findings described in their manuscript fully available without restriction, with rare exception (please refer to the Data Availability Statement in the manuscript PDF file). The data should be provided as part of the manuscript or its supporting information, or deposited to a public repository. For example, in addition to summary statistics, the data points behind means, medians and variance measures should be available. If there are restrictions on publicly sharing data—e.g. participant privacy or use of data from a third party—those must be specified.

Reviewer #1: Yes

Reviewer #2: Yes

---

4. Is the manuscript presented in an intelligible fashion and written in standard English?

PLOS ONE does not copyedit accepted manuscripts, so the language in submitted articles must be clear, correct, and unambiguous. Any typographical or grammatical errors should be corrected at revision, so please note any specific errors here.

Reviewer #1: No

Reviewer #2: No

---

5. Review Comments to the Author

Please use the space provided to explain your answers to the questions above. You may also include additional comments for the author, including concerns about dual publication, research ethics, or publication ethics. (Please upload your review as an attachment if it exceeds 20,000 characters)

Reviewer #1: The research question has been widely addressed by recent published literature and fails to add to the existing knowledge base. Examples include:
Pitanupong J, Sathaporn K, Ittasakul P, Karawekpanyawong N. Relationship of mental health and burnout with empathy among medical students in Thailand: A multicenter cross-sectional study. Plos One. 2023 Jan 5;18(1):e0279564.
Picard J, Catu-Pinault A, Boujut E, Botella M, Jaury P, Zenasni F. Burnout, empathy and their relationships: a qualitative study with residents in General Medicine. Psychology, health & medicine. 2016 Apr 2;21(3):354-61.
Thirioux B, Birault F, Jaafari N. Empathy is a protective factor of burnout in physicians: new neuro-phenomenological hypotheses regarding empathy and sympathy in care relationship. Frontiers in psychology. 2016 May 26;7:763.

Amendments were made in the introduction section in order to satisfy the request of the reviewer's comment.

Reviewer #2: Dear Author, Though it is an interesting research, there is always room for improvement. You may kindly consider the following points:
1) Thoroughly revise your manuscript and read your Abstract at two places in your article and make sure that both texts are the same and present in the main article as well. Try to improve your wording.

The abstract was reviewed and revised.

2)Give further strength to your rationale/purpose of study.

The rationale and purpose of the study were reviewed and completed.

3)In methods, too much detail is given under MEASURES section. Try to add your own performed METHODS rather than giving a literature review. Consider renaming your study design. Mention type of regression used. Exclusion criteria has some not-included variables which may be deleted. If it is a census then there is no need to use the word SAPMLE or sampling technique.

In the method section, corrections were made according to the reviewer's comment, and the manuscript was corrected based on the reviewer’s comments.

4) Reconsider your tables and make it more comprehensible and in standard formats especially table 1 and table 4. Table 3 can be adjusted within table 4. Kindly get guidance from other published researches how to report regression analysis.

The tables were revised and Table 3 was merged into Table 4.

5) Rewrite a short but strong conclusion keeping in view your study objective and results.

The conclusion was reviewed and revised in the abstract and in the final paragraph of the manuscript.
