# Supplementary material for: Association between burnout and empathy in medical residents
Source: PLoS One. 2024 Apr 9;19(4):e0301636. doi: 10.1371/journal.pone.0301636 (PMC11003688; doi:10.1371/journal.pone.0301636)
Supplement: Attachment — Submitted filename: Response to Reviewers.docx [file pone.0301636.s001.docx]

**Dear Dr. El Seifi,
Academic Editor PLOS ONE**

*PONE-D-23-22729. Association between Burnout and Empathy in Medical Residents*


It is with great pleasure that we resubmit to you a revised version of manuscript entitled "*Association between Burnout and Empathy in Medical Residents*". We would like to thank you for giving us the opportunity to revise and resubmit this manuscript. We appreciate the time and care you and the reviewers took in providing us with the valuable comments, and we have incorporated the suggested changes into the manuscript to the best of our ability. No doubt that these insightful comments have added to the theoretical and practical strength of this manuscript. We are ready to take more suggestions from you should you think the revised manuscript is still in need of further refinement.

Kind regards,

**Corresponding author**

**Abdolhussein Shakurnia**

==============================
ACADEMIC EDITOR:

- Please provide strong justification of the importance of your research and difference between it and other similar studies specially in your country.

According to the reviewer's comment, corrections were made in the final paragraph of the introduction section.

- The study design: it's known that the observational correlational studies include; cross sectional studies, case control studies, cohort studies. As your study is suitable to be crooss sectional study design, so I prefere to be more specific in mentioning the study design as cross section study.

In the manuscript, the study design was to cross-sectional.

- The method part need to be more oraganized as; study design, timing and setting, participant, sampling, tools of data collection, ethical consideration and data mangment and analysis.

The method section was organized according to the reviewer's comment.

- Please mention the response rate of the participants in the method section.

According to the reviewer recommendation, the response rate was transferred to the method section.

- Did you conduct a pilot study?

No

==============================


Journal Requirements:

When submitting your revision, we need you to address these additional requirements.

1. Please ensure that your manuscript meets PLOS ONE's style requirements, including those for file naming. The PLOS ONE style templates can be found at

https://journals.plos.org/plosone/s/file?id=wjVg/PLOSOne_formatting_sample_main_body.pdf and

<https://journals.plos.org/plosone/s/file?id=ba62/PLOSOne_formatting_sample_title_authors_affiliations.pdf>.

Our manuscript meets PLOS ONE's style requirements, including those for file naming. The PLOS ONE style templates.

2. Please provide additional details regarding participant consent. In the ethics statement in the Methods and online submission information, please ensure that you have specified what type you obtained (for instance, written or verbal, and if verbal, how it was documented and witnessed). If your study included minors, state whether you obtained consent from parents or guardians. If the need for consent was waived by the ethics committee, please include this information.

The data were collected in a hospital following relevant guidelines and using a paper-based process. As far as the paper-based method was concerned, a research assistant handed the participants an information sheet delineating the study's rationale and the allotted time to complete the survey. They had 10 minutes to decide whether they were willing to collaborate in the study or not. If they were, the research assistant would hand them the questionnaires. Participation in the study was voluntary, and there was no compensation for completing the questionnaire. Adhering to the policy of strict confidentiality, the signatures of the participants were not required, and all the participants retained the right to withdraw from the research at any time without giving any reason.

3. In your Data Availability statement, you have not specified where the minimal data set underlying the results described in your manuscript can be found. PLOS defines a study's minimal data set as the underlying data used to reach the conclusions drawn in the manuscript and any additional data required to replicate the reported study findings in their entirety. All PLOS journals require that the minimal data set be made fully available. For more information about our data policy, please see <http://journals.plos.org/plosone/s/data-availability>.

All data are available by corresponding author via Email ([shakurnia@yahoo.com](mailto:shakurnia@yahoo.com)) upon reasonable request.

"Upon re-submitting your revised manuscript, please upload your study’s minimal underlying data set as either Supporting Information files or to a stable, public repository and include the relevant URLs, DOIs, or accession numbers within your revised cover letter. For a list of acceptable repositories, please see http://journals.plos.org/plosone/s/data-availability#loc-recommended-repositories. Any potentially identifying patient information must be fully anonymized.

Important: If there are ethical or legal restrictions to sharing your data publicly, please explain these restrictions in detail. Please see our guidelines for more information on what we consider unacceptable restrictions to publicly sharing data: http://journals.plos.org/plosone/s/data-availability#loc-unacceptable-data-access-restrictions. Note that it is not acceptable for the authors to be the sole named individuals responsible for ensuring data access.

We will update your Data Availability statement to reflect the information you provide in your cover letter.


Reviewers' comments:

Reviewer's Responses to Questions

**Comments to the Author**

1. Is the manuscript technically sound, and do the data support the conclusions?

The manuscript must describe a technically sound piece of scientific research with data that supports the conclusions. Experiments must have been conducted rigorously, with appropriate controls, replication, and sample sizes. The conclusions must be drawn appropriately based on the data presented.

Reviewer #1: Partly

Reviewer #2: Partly

2. Has the statistical analysis been performed appropriately and rigorously?

Reviewer #1: Yes

Reviewer #2: Yes

3. Have the authors made all data underlying the findings in their manuscript fully available?

The [PLOS Data policy](http://www.plosone.org/static/policies.action#sharing) requires authors to make all data underlying the findings described in their manuscript fully available without restriction, with rare exception (please refer to the Data Availability Statement in the manuscript PDF file). The data should be provided as part of the manuscript or its supporting information, or deposited to a public repository. For example, in addition to summary statistics, the data points behind means, medians and variance measures should be available. If there are restrictions on publicly sharing data—e.g. participant privacy or use of data from a third party—those must be specified.

Reviewer #1: Yes

Reviewer #2: Yes

4. Is the manuscript presented in an intelligible fashion and written in standard English?

PLOS ONE does not copyedit accepted manuscripts, so the language in submitted articles must be clear, correct, and unambiguous. Any typographical or grammatical errors should be corrected at revision, so please note any specific errors here.

Reviewer #1: No

Reviewer #2: No

5. Review Comments to the Author

Please use the space provided to explain your answers to the questions above. You may also include additional comments for the author, including concerns about dual publication, research ethics, or publication ethics. (Please upload your review as an attachment if it exceeds 20,000 characters)

Reviewer #1: The research question has been widely addressed by recent published literature and fails to add to the existing knowledge base. Examples include:
Pitanupong J, Sathaporn K, Ittasakul P, Karawekpanyawong N. Relationship of mental health and burnout with empathy among medical students in Thailand: A multicenter cross-sectional study. Plos One. 2023 Jan 5;18(1):e0279564.
Picard J, Catu-Pinault A, Boujut E, Botella M, Jaury P, Zenasni F. Burnout, empathy and their relationships: a qualitative study with residents in General Medicine. Psychology, health & medicine. 2016 Apr 2;21(3):354-61.
Thirioux B, Birault F, Jaafari N. Empathy is a protective factor of burnout in physicians: new neuro-phenomenological hypotheses regarding empathy and sympathy in care relationship. Frontiers in psychology. 2016 May 26;7:763.

Amendments were made in the introduction section in order to satisfy the request of the reviewer's comment.

Reviewer #2: Dear Author, Though it is an interesting research, there is always room for improvement. You may kindly consider the following points:
1) Thoroughly revise your manuscript and read your Abstract at two places in your article and make sure that both texts are the same and present in the main article as well. Try to improve your wording.

The abstract was reviewed and revised.

2)Give further strength to your rationale/purpose of study.

The rationale and purpose of the study were reviewed and completed.

3)In methods, too much detail is given under MEASURES section. Try to add your own performed METHODS rather than giving a literature review. Consider renaming your study design. Mention type of regression used. Exclusion criteria has some not-included variables which may be deleted. If it is a census then there is no need to use the word SAPMLE or sampling technique.

In the method section, corrections were made according to the reviewer's comment, and the manuscript was corrected based on the reviewer’s comments.

4) Reconsider your tables and make it more comprehensible and in standard formats especially table 1 and table 4. Table 3 can be adjusted within table 4. Kindly get guidance from other published researches how to report regression analysis.

The tables were revised and Table 3 was merged into Table 4.

5) Rewrite a short but strong conclusion keeping in view your study objective and results.

The conclusion was reviewed and revised in the abstract and in the final paragraph of the manuscript.
