# Supplementary material for: Association between burnout and empathy in medical residents
Source: PLoS One. 2024 Apr 9;19(4):e0301636. doi: 10.1371/journal.pone.0301636 (PMC11003688; doi:10.1371/journal.pone.0301636)
Supplement: Attachment — Submitted filename: Response to Reviewers.docx [file pone.0301636.s003.docx]

Dear Omnia Samir El Seifi, M.D.
Academic Editor PLOS ONE,

PONE-D-23-22729. Association between Burnout and Empathy in Medical Residents

It is with great pleasure that we resubmit to you a revised version of manuscript entitled "Association between Burnout and Empathy in Medical Residents". We would like to thank you for giving us the opportunity to revise and resubmit this manuscript. We appreciate the time and care you and the reviewers took in providing us with the valuable comments, and we have incorporated the suggested changes into the manuscript to the best of our ability.

The edited file of the manuscript is sent to you in two forms, a clean and Revised Manuscript with Track Changes.

The reference list was checked. The correctness of the reference is confirmed.

With regards,

Corresponding author

Abdolhussein Shakurnia
